# Metal–organic frameworks (MOFs) for nonlinear optical properties: design principles, DFT insights, and future directions for photonic applications

**DOI:** 10.1039/d6ra03903k

**Published:** 2026-07-09

**Authors:** Sehar Nadeem, Muhammad Usman Khan

**Affiliations:** a Department of Chemistry, University of Okara Okara-56300 Pakistan usman.chemistry@gmail.com usmankhan@uo.edu.pk

## Abstract

Metal–organic frameworks (MOFs) have emerged as a uniquely versatile platform for nonlinear optical (NLO) applications, combining the large hyperpolarizabilities of organic chromophores with the chemical robustness and structural programmability of crystalline porous materials. Although many reviews have covered various facets of MOF-NLO chemistry, no review has brought together structural design principles, family-to-family quantitative performance comparisons, and DFT-based hyperpolarizability evaluations within a unified critical framework. This review of NLO properties of MOFs is considered through three interconnected facets: the principles of structural design to achieve non-centrosymmetric, high-second and third-order response structures; computational tools based on density functional theory (DFT) to predict and rationalize the electronic and optical properties of MOFs; and the measurement tools such as Z-scan and two-photon excited fluorescence (TPEF). The particular focus is on triphenylamine (TPA)-based MOFs, with their multi-branched donor–π–acceptor structure and robust intramolecular charge transfer (ICT) features rendering them the most promising third-order NLO materials. They provide externally tunable third-order responses (*β* = 10^−3^ to 10^−4^ cm W^−1^) *via* electric-field modulation, guest loading, and interpenetration engineering. Moreover, the MOF families lanthanide MOFs, bimetallic Zn/Cu systems, Ti-based MIL-125, Zr-based UiO-66, Cu-HHTP, bismuth-organic frameworks, zeolitic imidazolate frameworks, and porphyrin-based 2D frameworks are critically considered based on their NLO activity. Second-order NLO activity (*d*_33_ ≈ 19.86 pm V^−1^, ∼12× KDP) is maximized using bimetallic Zn/Cu MOFs and electrically tunable Cu-HHTP films, which represent the state-of-the-art for actuatable switched third-order polymeric NLO materials. The DFT methods, such as hybrid functionals, dispersion-corrected methods and machine learning-accelerated screening emerging methods, are evaluated based on their ability to quantitatively predict band gaps, charge-transfer energies, and hyperpolarizability tensors. High-priority research frontiers include frequency-dependent NLO computations, chiral MOF engineering for SHG, guest@MOF switching, and 2D nanosheet architectures. This review provides not only a practical design guide but also a significant computational roadmap for emerging MOF-based photonic technologies such as optical data storage, ultra-fast all-optical switching, frequency-conversion lasers, and non-invasive bio-imaging.

## Abstract 2

A critical review of NLO properties in MOFs, covering structural design, DFT insights, and experimental characterization, with focus on triphenylamine-based systems and emerging computational strategies for photonic applications.

## Graphical abstract

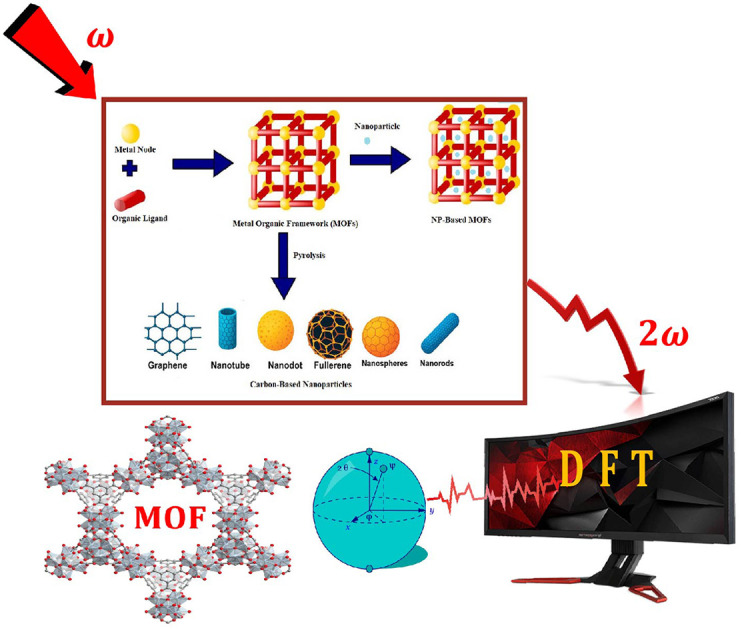

## 1. Introduction

Nonlinear optical phenomena arise when the optical response of a material ceases to scale linearly with the incident electric field, a regime accessible only with the high photon fluxes provided by pulsed laser sources.^1–3^ The resulting effects, such as second-harmonic generation (SHG), two-photon absorption (TPA), third-harmonic generation (THG), and optical limiting, underpin technologies spanning frequency conversion, ultrafast all-optical switching, three-dimensional data storage, and bio-imaging with near-infrared excitation.^4–6^ The practical utility of any NLO material is governed by two largely independent sets of properties: the magnitude of the nonlinear susceptibility (*χ*^(2)^ or *χ*^(3)^ at the macroscopic level; hyperpolarizabilities *β* and *γ* at the molecular level) and the chemical robustness to sustain repeated laser exposure without degradation.^7,8^ Decades of research have established that no single material class satisfies both requirements simultaneously, motivating the ongoing search for structurally programmable hybrid systems. Two-photon absorption cross-sections (*σ*_2_) of more than 104 GM (Göppert-Mayer, 10^−50^ cm^4^ per s per photon) and first hyperpolarizabilities several orders of magnitude greater than that of benchmark inorganic crystals such as potassium dihydrogen phosphate (KDP) or quartz can be achieved with organic chromophores, in particular push–pull π-conjugated systems with donor and acceptor groups separated in space.^9,10^ The underlying physics is straightforward: intramolecular charge transfer (ICT) along an extended π-system leads to a large change in dipole moment upon excitation, which directly enhances the nonlinear polarization.^9^ The problem is that these same attributes in long π-conjugation, amino-based electron donors, flexible backbones, also lead to poor photochemical and thermal stability, concentration-dependent fluorescence quenching in the solid state, and poor film-forming properties.^6,11^ Inorganic materials (quantum dots, plasmonic nanoparticles, and lanthanide-based nanocrystals) resist photobleaching better but lose molecular-level tunability; their NLO responses are dominated by band-gap physics and crystal-field effects, offering the chemist little synthetic control for rational design.^12,13^ MOFs are structurally unique in this regard. MOFs are comprised of metal ions or polynuclear metal clusters, commonly referred to as secondary building units (SBUs), bridged by multitopic organic linkers to form a periodic, porous material.^14,15^ Coordination immobilizes the linker in the crystal lattice, thereby removing the conformational and rotational degrees of freedom that are responsible for non-radiative decay and concentration quenching in solution-phase chromophores.^16,17^ Ligand-to-metal or metal-to-ligand charge transfer (LMCT/MLCT) transitions, which result from coordination, also modulate frontier orbital energies in ways that can enhance polarizability and hyperpolarizability beyond the contributions from the free linker.^18,19^ Intermolecular cooperative effects, such as π–π interactions between adjacent chromophore units, delocalization of excitons across the crystal lattice, and crystal packing-induced planarity of otherwise non-planar linkers, also affect the macroscopic NLO response in a manner that cannot be predicted by a simple summation of the individual contributions from the linkers.^20,21^

Recent developments in nanocomposite engineering have also expanded the range of NLO materials, with MOFs hybridized with quantum dots of perovskite structures, reduced graphene oxide, and plasmonic nanoparticles exhibiting synergistic enhancement of NLO properties that exceeds those of individual components.^22–24^ In general, the optical properties of hybrid nanocomposite systems, such as polymer matrices doped with metal oxide nanofillers (TiO_2_, Fe_2_O_3_ and Co_3_O_4_), have been found to exhibit tunable optical band gaps, high dielectric constants and multifunctional optoelectronic responses and continue to provide inspiration for MOF-based NLO platforms.^25–28^

First, MOFs are opted for this review owing to the following five advantages over traditional NLO materials. Inorganic and organic components can be tuned independently, based on their modular reticular chemistry, offering rational control of the chemical characteristics of the systems (band gap, charge-transfer energy and hyperpolarizability), which is not available in purely organic or inorganic systems.^29^ Second, chromophores in the solution phase with MOF are conformationally disordered, and therefore experience concentration quenching for their NLO performance.^16^ Third, the presence of a periodic crystal framework facilitates cross-wise π stacking mechanisms for propagation of NLO through molecule–molecule interactions and lateral spreading of the excited state throughout the crystal; each process likely contributing to an increased macro-level NLO response in the ensemble when compared to that observed for individual molecules^30^ from all material classes competitors to nitro organized class.^31^ The fifth is high-valency nodes we observe in the Zr_6_ cluster of UiO-66, a release-unresponsive framework-dense chromophore that provides great photochemical resistance upon repetitive laser exposure not possible through common organic dyes.^32^

The modular reticular chemistry of MOFs is particularly optimized by NLO.^33^ This design rationale can be extended into non-centrosymmetric space groups needed to enable non-monolithic activity (*χ*^(2)^ ≠ 0), and can be extended in various ways to provide a donor–acceptor asymmetry by replacing functional groups, control framework dimensionality and interpenetration degree, and exploit pores geometry to define NLO-active guest molecules because the geometry of the frontier unit (SBU).^18,34^ However, a large practical issue is that most MOFs are centrosymmetrically crystallized, preclinically unsuitable for SHG because of centrosymmetry, in particular centrosymmetrically crystallized using symmetric aromatic dicarboxylate linkers.^35,36^ Hence one of the potential pathways to solving a synthetic problem in the area is to arrive at non-centrosymmetric structures through the design of linkers chiral, self-assembly, template design, ornselective choice of metal nodes.^37,38^

Despite this common advancement, four important gaps appear in the current MOF-NLO literature. This stems first from the fact that most studies published today report NLO parameters for single MOF systems in a vacuum without comparing NLO parameters across their class of framework type, bringing some complexity to the reader as they will not leave knowing which framework family yields best performance for any given photonic application. Second, DFT calculations in most of the MOF-NLO papers are performed only at the static band gap and absorption onset level using standard GGA functionals which systematically underpredict band gaps to 1–2 eV and therefore overpredict hyperpolarizability. Yet very few studies benchmark experimental Z-scan or SHG measurements against their computational predictions.^39^ Finally, there is a dearth of frequency-dependent hyperpolarizability calculations at experimentally relevant laser wavelengths (532 nm, 1064 nm), the workhorses in molecular NLO computation in the MOF-NLO literature that creates an almost imperceptible rift between theory and experiment that hinders rational design.^40^ Fourth, TPA-based MOFs, they show some of the strongest third-order NLO responses of any material ever reported that have not been comprehensively reviewed as a distinct physical subclass and the external control strategies that render them particularly advantageous candidates for active photonic devices have yet to be systematically assessed.^41^ This review tackles each of these deficiencies head-on by providing the assessment, theoretical background and design principles necessary to translate MOF–NLO materials from a proof-of-concept phase into viable photonic devices.

NLO properties of MOFs and coordination polymers had already be treated in previous review articles. Wang *et al.*,^18^ provided pioneering structure–property relationships for second-order NLO MOFs but preceded the development TPA-based frameworks, conductive MOF films and ML-accelerated screening. Medishetty *et al.*,^20^ report review on multiphoton harvesting and lasing in MOFs which covers many aspects of this area but does not consider methods for DFT hyperpolarizability calculations or frequency-dependent NLO methods. Mingabudinova *et al.*,^42^ has extensively surveyed MOFs as potential NLO materials with particular emphasis on SHG or the context of metamaterial applications but without a systematic quantitative analysis across families that allows for performance comparisons against benchmark inorganic crystals. In contrast, the present review is distinct from these predecessors at three levels: (i) we provide the first systematic quantitative comparison of NLO performance across all major MOF families using unified benchmarks for *β*, *χ*^(3)^, *d*_33_ with cited references to KDP and α-quartz; (ii) the identification and critical assessment of TPA-based MOFs as a specific high-performing subclass with detailed treatment of modulation strategies unavailable in any previous review; (iii) an evaluation of DFT-based hyperpolarizability calculation methods including frequency-dependent TD-DFT and machine learning acceleration. A computational roadmap absent from all prior MOF-NLO reviews.

Despite these obstacles, the last ten years yielded enough proof-of-concept progress. Quah, Vittal and coworkers demonstrated multi-photon (two-, three-, and four-photon) absorption in pillared-layer zinc MOFs, establishing that solid-state multiphoton cross-sections could be extracted reliably using modified excited fluorescence protocols.^43^ Medishetty *et al.*, subsequently reported stimulated emission from indium- and zinc-MOFs built on the TCPE linker, where the rigid, densely packed chromophore environment lowered the threshold pump fluence markedly relative to dye solutions.^44^ On the second-order side, Evans and Lin established early structure–property relationships for diamonded and helical CPs, demonstrating that SHG efficiencies up to several hundred times that of α-quartz were accessible by systematic ligand elongation.^45,46^

**Fig. 1:**
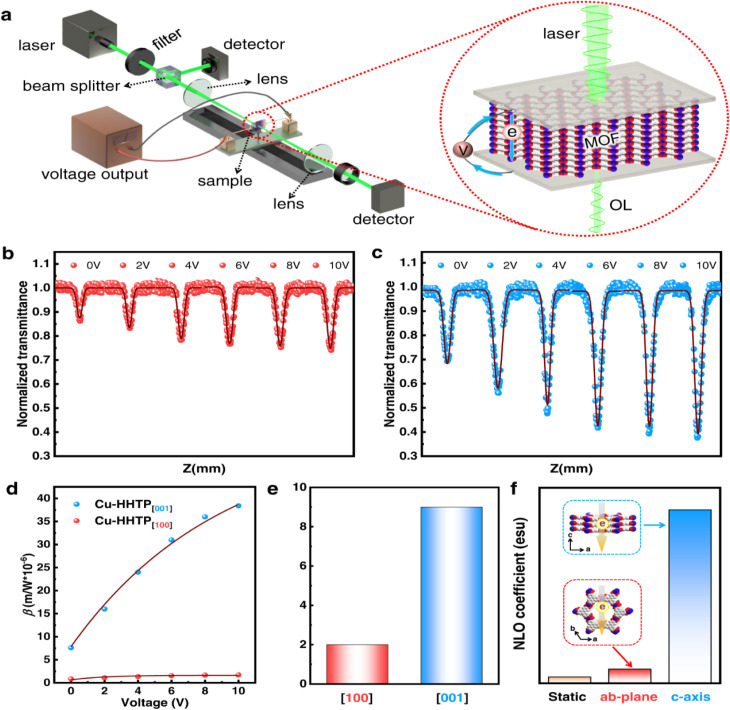
Electrically tunable NLO properties of Cu-HHTP films studied by the Z-scan and theoretically modeled. (a) Experimental Z-scan system with a built-in external power supply. (b) and (c) Open-aperture Z-scan results of Cu-HHTP [001] and Cu-HHTP [100] films at various voltages. (d) and (e) Evolution of the nonlinear absorption coefficient (*β*), and *R* values of both orientations, dependent on voltage. (f) Comparison of calculated third order polarizabilities (*γ*) along the *c*-axis and the *ab*-plane in the presence of applied electric fields, and the electron transport model of Cu-HHTP. Reproduced from ref. 31 with permission from Springer Nature, Z.-Z. Ma, *et al.*, *Nat. Commun.*, 2022, **13**, 6347, copyright 2022.

The TPA unit, which consists of three equivalent electron-donating arms in propeller shape, has recently become of particular interest as a building block in an NLO-active MOF. These systems have since been estimated using hybrid functionalities with dispersion corrections to achieve high SHG efficiencies exceeding the KDP standard, *χ*^(3)^ = 10^−7^ esu as shown in Fig. 1 and a range of electrically controllable NLO response.^31,47^ Simultaneously, DFT computations with dispersion corrections of hybrid functionalities have been exercised to these systems to correlate a linker substitution pattern with calculated band gaps, charge-transfer energies and hyperpolarizability tensors, which furnish a computational foundation to understand experimental trends.^48,49^

There are three themes reviewed. The former is structural design – namely, how non-centrosymmetric MOFs with high second-order responses are prepared, focusing on linker geometry, choice of metal node, dimensionality and interpenetration.^35^ Second, we consider TPA-derived MOFs as a model of how donor-engineered linkers can be converted into macroscopic NLO activity, and how external conditions, such as electric field, guest loading, redox switching, can be reversibly changed into such activity.^47^ Third, we evaluate the application of DFT-based computational methods, which are being extended by machine learning-aided screening, to predict and understand NLO properties in MOF families, and the quantitative limits of current theoretical methods.^50^ We also compare and contrast the measurement methods, Z-scan and two-photon excited fluorescence (TPEF), and their solid-state analogs in the determination of the NLO properties, and critically assess the challenges that have been faced in applying the methods to crystalline, poorly soluble, or light-scattering MOF samples.^51,52^ We do not intend to create an exhaustive list of reported NLO-active MOFs, but rather to critically examine the design principles, measurement standards, and theoretical foundations required to translate these materials out-of-concept demonstrations into practice applications of photonics.

## 2. Theoretical perspective of non-linear optical material

NLO occurs when the behavior of a substance to an incident electromagnetic field becomes dependent on the strength of the incident light.^53^ Once a material is irradiated to high-intensity laser, the induced polarization (*P*) of the material stops behaving linearly and can be written as a power series of the electric field (*E*):^53^

1 *P* = *ε*_0_(*χ*^(1)^*E* + *χ*^(2)^*E* + *χ*^(3)^*E* + …)

Vacuum permittivity is donated by *ε*_0_, *χ*^(3)^, *χ*^(2)^, *χ*^(1)^ are the third-order nonlinear, second-order, linear susceptibilities, respectively. The higher orders represent the non-linear response and linear term represent the low intensity and ordinary linear phenomena.^17^ Second-harmonic generation (SHG) and sum-frequency generation are processes that are governed by second-order nonlinear effects, which are described by *χ*^(2)^. These effects depend on the symmetry of the material immensely and can only occur in non-centrosymmetric systems.^54,55^ Conversely, all materials have third-order nonlinear processes, expressed as *χ*^(3)^, and thus such processes are especially important in the context of metalorganic frameworks, which are generally centrosymmetric.^17,54^ In practical application, two factors are important non-linear refraction and nonlinear absorption. Two-photon absorption (TPA) and multi-photon absorption are examples of non-linear absorption, in which two or more photons are absorbed simultaneously to cause a molecule to transition out of the ground state to an excited state. Instead, nonlinear refraction is due to the dependence of changes in the refractive index on intensity and is typically explained by the equation:

2 *n* = *n*_0_ + *n*_2_*I*

*n*_0_ and *n*_2_ are the co-efficient for linear and non-linear refractive index and *I* is the instantaneous optical intensity of the incident laser beam (units: W cm^−2^). A positive *n*_2_ corresponds to self-focusing behavior while a negative *n*_2_ corresponds to self-defocusing behavior.

These third order effects are key to applications like optical limiting, all optical switching, and photonic modulation.^16,42^ At the molecular level, the NLO response of the material is closely in relation to the redistribution and polarization of the system under an applied electric field. Here long π-conjugation, and rigorous intramolecular charge transfer (ICT) play major roles to increase nonlinear susceptibilities.^18,36^ Particularly in donor acceptor (D) systems have very large changes in the dipole moment when excited, which makes the systems to generate large third-order responses.^18^ Such molecular features can now be included in ordered structures, *e.g.* MOFs to be enabled to translate microscopic nonlinearities into macroscopic optical behavior.^56^ In metal–organic frameworks, the nonlinear optical characteristics of the structure are determined by the complex interaction of organic ligands, metal nodes and the topological structure of the framework. Their electronic structure is controlled by coordination of ligands to metal centers, such as metal-to-ligand (MLCT) and ligand-to-metal (LMCT) charge transfer interactions that can also further increase polarizability.^33^ The periodic arrangement of chromophore units into the crystal lattice also allows cooperative contributions like π–π stacking of the units and exciton delocalization, which are well-known to enhance nonlinear response.^56^ In the case of triphenylamine-based MOFs, such effects are strongly enhanced by the fact that the TPA core is an excellent electron-donor, and can readily form multi-branched conjugated systems. The combination of TPA like donor activity and electron-withdrawing coordination groups in MOFs results in effective charge-transfer effects, and thus, greatly boosts third-order nonlinear optical reactions.^18,19^ Moreover, with these systems arranged in two-dimensional structures, the corresponding anisotropy and increased form of electronic interaction can develop better interaction of light and matter and adjustable optical characteristics.^55^

## 3. Techniques to determine the nonlinear optical properties of MOFs

To accurately determine the structure properties relationship and assess the appropriateness of the metalorganic framework (MOF) to be used in photonic applications, the characterization of NLO properties is crucial in metalorganic frameworks (MOFs).^12,57,58^ Of the several available experimental methods the most popular ones used to probe the third order non-linearities such as non-linear absorption and non-linear refraction are the Z-scan and the two-photon excited fluorescence (TPEF).^57,59^ Of special interest is the Z-scan method, which has now become a more or less standard because it is relatively simple and can measure both the nonlinear absorption coefficient (*n*_0_) and the nonlinear refractive index (*n*_2_). The sample in this approach is conveyed in the direction of propagation of a focused laser beam and the sample undergoes different levels of light intensities about the focus point. The transmitted intensity changes are first measured in an open-aperture setup, using which one can know about non-linear absorption mechanisms, such as two-photon absorption and reverse saturable absorption, and in a closed-aperture setup, or which is sensitive to nonlinear refraction effects.^57,60^ Z-scan data can be analyzed with the help of the fitting of the experimental transmittance curves to theoretical models that allow the derivation of the essential NLO parameters, but the accuracy of such approach highly relies on the accurate awareness of the laser beam properties, such as the intensity distribution and the pulse length.^57^

Although it has been extensively used, the utilization of Z-scan measurements to MOFs is associated with various challenges that mainly occur because of the physical nature of these materials. The majority of MOFs are collected as crystalline powders or microcrystalline solids and are both likely to scatter light when dispersed in solvents, thus giving rise to distortions in the measured signal.^12,58^ Also, the uncertainties can be caused by sedimentation and uneven distribution of particles. Moreover, the use of care should be taken to differentiate between the true multiphoton absorption and other competing processes like excited-state absorption, thermal effects, as well as nonlinear scattering that may also lead to the observed nonlinear response.^58,60^ Z-scan method was used to assess the third-order nonlinear optical (NLO) properties of the sample. A pulsed Nd:YAG laser that had a repetition rate of 5 Hz, pulse duration of 5 ns, and a wavelength of 532 nm was used as the excitation light source. A beam splitter mirror was used to divide the laser beam into two directions and the pulse energy at the front and rear of the sample was measured simultaneously on two calibrated energy detectors (D1 and D2) thus providing real-time normalization of the signal sent. All measurements were conducted at ambient conditions and at room temperature. The sample was placed on a computer-controlled translation stage and the samples were systematically moved along the *z*-axis by the focal point of the beam. The Z-scan curves of the open-aperture were made into normalized transmittance *versus* input laser intensity of a spatially Gaussian beam. The light fluence *F*_in_(*z*) at any point *z* along the propagation axis was obtained by dividing the input laser pulse energy *E*_in_ and the beam radius *ω*(*z*).^31^*F*_in_(*z*) is defined as:
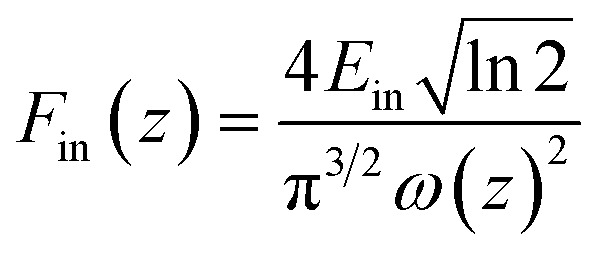


In above equation, *ω*(*z*) is defined as;
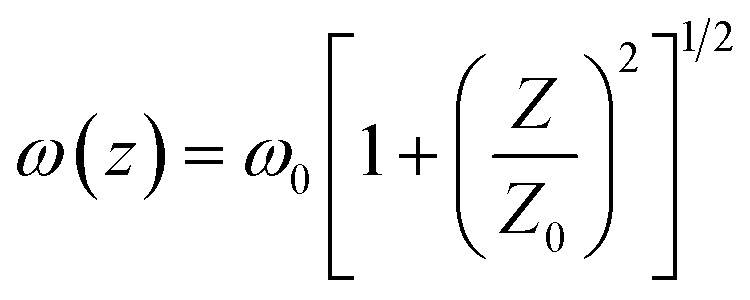


The Rayleigh range and light beam radius are represented by *z*_0_ and *ω*_0_ (29 µm)
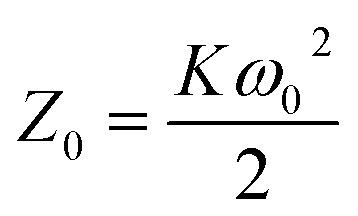
where *K* is equal to
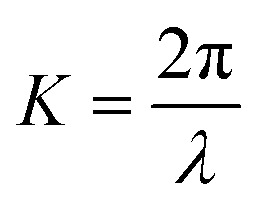

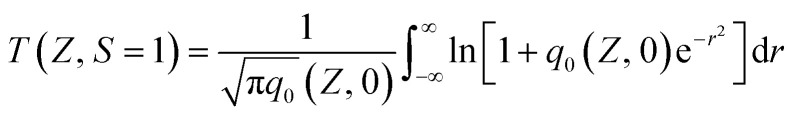
*q*_0_(*Z*, 0) = *βI*_0_*L*_eff_
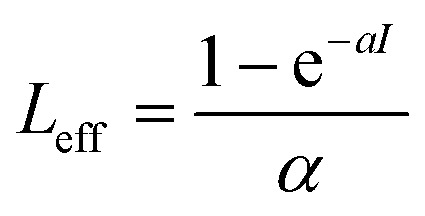
*I* donates the sample thickness, *α* represents the linear absorption coefficient, *L*_eff_ gives the effective thickness of the sample, and at the focus (*Z* = 0), *I*_0_ is the on-axis peak intensity.^31^

Another well-known application of the technique to measurement of two-photon absorption cross-sections (*σ*_2_) is two-photon excited fluorescence (TPEF), which is used to measure luminescent systems.^59^ Under this technique, the intensity of the generated fluorescence by the concurrent absorption of two photons is measured and the result compared to that of a reference compound with known characteristics. The TPEF signal is proportional to the intensity of the squared excitation intensity, and the quantum yield of fluorescence, so is also a sensitive probe of the nonlinear absorption process. The implementation of TPEF to MOFs, however, just like in the case of Z-scan, is made challenging by the scattering effect and variation in light collection efficiency between samples and reference solutions.^58^ To overcome these drawbacks, alternative methods like internal standard two-photon excited fluorescence (ISTPEF) have been introduced, whereby the MOF sample is measured with a reference dye and whereby the comparison is more reliable with regards to minimizing the differences in the scattering and optical geometry.^61^

Besides these main modes, other spectroscopic methods, including the transient absorption spectroscopy, and pump probe measurements are sometimes used as a means of understanding dynamics of excited states, and to differentiate between instantaneous and sequential nonlinear processes.^62^ Such techniques have most clearly been employed to study processes like excited-state absorption and energy transfer which are instrumental in defining the overall NLO response of MOFs. Their use however is frequently restricted to well dispersed nanoparticle systems or thin films because of the need to previously have optically clear samples.

In general, nonlinear optical properties of MOFs are to be studied accurately only with the proper choice of measurement techniques and experimental conditions (see Table 1). Laser wavelength, pulse duration, repetition rate, sample morphology and concentration, among others, have to be strictly regulated to achieve reliable and reproducible results.^57,58^ Such considerations, in particular, are relevant in the case of triphenylamine-based MOFs because the strength of charge-transfer effects and the structure of the framework have a significant effect on the observed NLO behavior.

**Table 1:** Third- and second-order nonlinear optical (NLO) properties of representative metal–organic frameworks (MOFs) obtained from degenerate four-wave mixing (DFWM), two-photon excited fluorescence (TPEF), Z-scan, multiphoton excited fluorescence (MPEF), and second-harmonic generation (SHG) techniques. Important parameters are nonlinear absorption coefficient (*β*, cm W^−1^), nonlinear refractive index (*n*_2_, cm^2^ W^−1^), third-order susceptibility (*χ*^(3)^, esu) and optical limiting threshold (J cm^−2^). SA = saturable absorption, RSA = reverse saturable absorption. All Z-scan measurements were done in 532 nm unless otherwise stated

No.	Technique	Conditions	MOF formula	NLO quantities	Ref.
1	[Z-scan]	Thin film, 532 nm, ns, 80 µJ	Cu_3_(HHTP)_2_ films: Cu-HHTP [001] and Cu-HHTP [100]	*β* = 7.60 × 10^−6^ m W^−1^ (Cu-HHTP [001]); 0.84 × 10^−6^ m W^−1^ (Cu-HHTP [100]) at 0 V	31
				*β* = 3.84 × 10^−5^ and 1.71 × 10^−6^ m W^−1^ at 10 V (electrically tunable)	
				*γ* (DFT) = 2.01 × 10^−31^ esu ([001], 10 V)	
2	[Z-scan]	Solid nanosheet/PDMS glass, 532 nm, ns	[Zn_2_(TPyP)(AC)_2_]_*n*_—ZnTPyP-1 (2-fold interpenetrated)	ZnTPyP-1: *β* = 3.61 × 10^6^ cm GW^−1^; *χ*^(3)^ = 7.73 × 10^−7^ esu	63
			[Zn_3_(TPyP)(C_2_O_4_)_2_(H_2_O)_2_]_*n*_—ZnTPyP-2 (non-interpenetrated)	OL threshold: 3.6 J cm^−2^ (ZnTPyP-1) *vs.* 11.7 J cm^−2^ (ZnTPyP-2)	
3	[Z-scan]	Solid state, 532 nm, ns	[Zn_3_(µ_3_-bta)_2_(µ_4_-ata)_2_]_*n*_—NH_2_–Zn-MUM-6	*n*_2_ = 30.83 × 10^−8^ cm^2^ W^−1^	64
				*β* = 4.06 × 10^−4^ cm W^−1^ (TPA, RSA regime)	
4	[Z-scan]	DMF dispersion, 532 nm, ns	Cu-bpy MOF ([Cu(bpy)]_*n*_, Cu(i), 4,4′-bipyridine)	*β* = 100 cm GW^−1^ (RSA)	65
				OL threshold = 0.75 J cm^−2^	
5	[Z-scan]	DMSO solution, CW, 532 nm	Cd-MOF: CdC_8_N_8_H_14_O_2_S_4_ (non-centrosymmetric)	*χ* ^(3)^ ∼ 10^−6^ esu (self-defocusing, RSA)	66
				OL threshold = 1094 kW cm^−2^	
6	[Z-scan]	Single crystal, gel-grown, CW 532 nm	SrCaEDTA bimetallic MOF (monoclinic, *C*2/*c*)	*χ* ^(3)^ exceeds KDP; strong RSA and self-defocusing (exact value not publicly tabulated)	67
7	[Z-scan]	Solid film, 532 nm, ns fs^−1^	Pillared Co-MOF (Co^2+^ + 4,4′-oxybis(benzoic acid) + amide linker; topology 3,4,5T94)	*n*_2_ = (6.6–19.1) × 10^−8^ cm^2^ W^−1^	68
				*β* = 2.92 cm GW^−1^ (RSA)	
8	[Z-scan]	Nanoplate suspension, CW, 532 nm, 30–50 mW	Mn-NBDC MOF (Mn^2+^ + 2-amino-1,4-benzenedicarboxylic acid)	*n*_2_ = 3.865 × 10^−10^ m^2^ W^−1^ (self-defocusing)	68
				*χ* ^(3)^ ∼ 10^−7^ esu; SA at *I*_0_ = 35 mW	
9	[Z-scan]	Thin film (LBL), 532 nm, ns	ZnTCPP(Zn)—2D homo-bimetallic porphyrin MOF thin film (LPE-LBL)	*χ* ^(3)^ ≈ 4.21 × 10^−7^ esu (highest in mono-/hetero-/homo-bimetallic series)	69
10	[Z-scan]	Thin film nanosheet array (LBL), 532 nm, ns	ZnTPyP(Co)—oriented 2D porphyrin MOF thin film (Co in porphyrin core)	*χ* ^(3)^ ≈ 2.63 × 10^−7^ esu (oriented nanosheet array + Co d–π conjugation)	[70]
11	[Z-scan]	Solid film, 532 nm, ns (defect engineering)	TCPP/UiO-66 and M(ii)-TCPP/UiO-66 (M = Co, Ni, Cu, Zn)—mixed-linker defect series	*β* and *n*_2_ greatly enhanced *vs.* pristine UiO-66; Co-TCPP/UiO-66 gives highest response	71
12	[Z-scan]	Solid film, 532 nm, ns	PANI@MIL-101(Cr)@CeO_2_ three-phase core–shell nanoarchitecture	*χ* ^(3)^ and *β* enhanced *vs.* bare MIL-101(Cr) *via* PANI and CeO_2_ shell synergy	24
13	[Z-scan]	Solid/dispersion, 532 nm, fs TAS + ns Z-scan	ABBr_3_-QDs@Cu-MOF (perovskite QDs in cage-like Cu-MOF; A = MA, FA; B = Pb, Sn)	Third-order NLO absorption enhanced ×6.36 *vs.* bare PeQDs; tuneable by cation composition	22
14	[Z-scan]	Solid state, 1064 nm, CW	Mg-MOF-74 (layered magnesium 2,5-dioxido-1,4-benzenedicarboxylate)	SA and RSA modes observed; specific *n*_2_, *β* in source	72
15	[Z-scan]	Sm-MOF hybrids, 532 nm ns + 700–1000 nm fs	Sm-MOF/rGO and Sm-MOF/BN hybrid composites	2PA (600 nm): *β* ∼ 10^−8^ m W^−1^	23
				3PA (700–1000 nm): *γ*_2_ ∼ 10^−20^ m^3^ W^−2^	
				Sm-MOF/rGO: best OL; BET = 276 m^2^ g^−1^	
16	[Z-scan]	PVA film (CP microcrystals), 532 nm, ns	[CdL(DMAc)(H_2_O)]_*n*_ (1) and {[CuL(4,4′-azobpy)]·3H_2_O}_*n*_ (2)—azobenzene-based CPs/PVA	SA → RSA switching with pulse energy; *β* photoswitchable *via trans* → *cis* azobenzene isomerization	73

**Third-order NLO—multiphoton excitation fluorescence/TPEF/MPEF (solid state)**					
17	[MPEF]	Solid state, 800–1450 nm, 110 fs	MOF-1 (perylene host MOF); anthracene@MOF-1 (MOF-2); perylene@MOF-1 (MOF-3)	*φσ*_2_ = 7.2 GM at 800 nm (2PA)	20
				*φσ*_3_ = 3.9 × 10^−79^ cm^6^ s^2^ at 950 nm (3PA)	
				*φσ*_4_ = 5.7 × 10^−110^ cm^8^ s^3^ at 1450 nm (4PA)	
				Guest encapsulation enhances *φσ*_*n*_*via* Förster resonance transfer	
18	[TPEF]	Solid state, 750 nm, fs	ZJU-23-Eu (2D layered centrosymmetric Eu-MOF)	ZJU-23-Eu: strong 2-/3-photon excited Eu^3+^ luminescence at 614 nm	74
			ZJU-24-Eu (3D non-centrosymmetric Eu-MOF)	ZJU-24-Eu: no TPEF (poor ligand stacking); SHG = 6.2× KDP at 1060 nm	
19	[TPEF]	Solid state, 860 nm and 1250 nm, fs	ZTIG (Zr-TCPP MOF + iridium complex); ZrTcI MOF	1, 2, 3-Photon CLSM bioimaging at 405, 860, 1250 nm; NIR-II imaging in HepG2 cells and myocardial tissue confirmed	75

**Second-order NLO—second-harmonic generation (SHG)**					
20	[SHG]	Crystal powder, 800–1500 nm, fs NIR	MOF-Er^3+^ (1) and codoped MOF-Yb^3+^/Er^3+^ (2)—noncentrosymmetric isostructural Ln-MOFs	Tunable SHG over 800–1500 nm; combined SHG + NIR Er^3+^ luminescence; used as NLO thermometer	76
21	[SHG]	Crystal powder, 800–1500 nm, fs NIR	MOF-Er^3+^ (1) and codoped MOF-Yb^3+^/Er^3+^ (2)—noncentrosymmetric isostructural Ln-MOFs	Tunable SHG over 800–1500 nm; combined SHG + NIR Er^3+^ luminescence; used as NLO thermometer	76

**Third-order NLO—DFWM and mixed techniques**					
22	[DFWM]	Solid state/solution, 800 nm, 80 fs, 1 kHz	Fe/Co/Mn/Zn-porphyrin coordination frameworks (Fe-THPP, Co-THPP, Mn-THPP, Zn-THPP)	*γ* = 1.42 × 10^−28^ to 7.64 × 10^−28^ esu (all four frameworks)	77
				RSA and self-focusing at 532 nm; best *γ* comparable to top porphyrin assemblies	
23	[Z-scan]	Solid powder, 800 nm, ∼70 fs, 1 kHz (OA + CA)	CTP-TAPB (imine-linked cyclotriphosphazene framework)	CTP-TAPB: *β* = −0.023 cm GW^−1^; *n*_2_ = −2.12 × 10^−7^ cm^2^ GW^−1^; *χ*^(3)^ = −9.92 × 10^−8^ esu	78
			CTP-TCPB (vinylene-linked cyclotriphosphazene framework)	CTP-TCPB: *β* = −0.019 cm GW^−1^; *n*_2_ = −1.62 × 10^−7^ cm^2^ GW^−1^; *χ*^(3)^ = −7.58 × 10^−8^ esu (negative values = SA + self-defocusing)	
24	[Z-scan]	ZnS/PPF-3 composite film, 532 nm, ns	ZnS@PPF-3 (ZnS nanoparticles *in situ* on porphyrin paddlewheel framework-3 surface)	*β* and *χ*^(3)^ enhanced *vs.* bare PPF-3 by ZnS loading; synergistic charge transfer	79
25	[Z-scan]	Solid state, fs broadband 700–950 nm, MHz	Prussian Blue nanoparticles (Fe_4_[Fe(CN)_6_]_3_·nH_2_O)—analogous MOF-type framework	3PA confirmed by pump-probe (fast ∼ pulse-length + slow ∼ 260 fs components); strong IR 3PA merit factor reported	20

## 4. Structural designing strategy

**Fig. 2:**
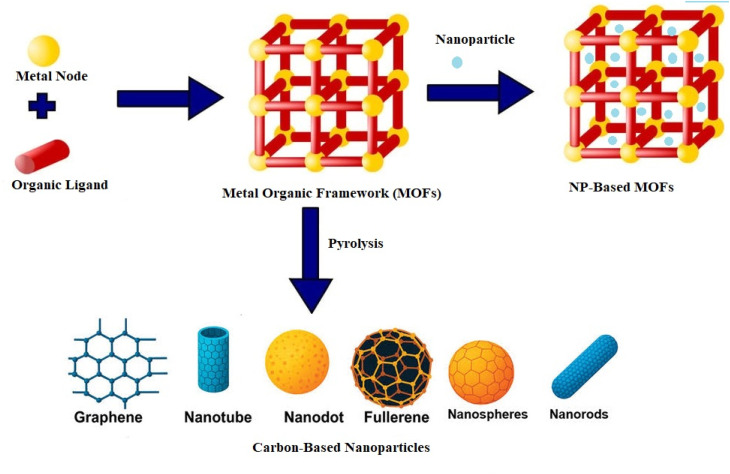
A general diagram MOFS, nanoparticle-based MOFs and carbon-based nanoparticles.

MOFs are self-assembled crystalline and porous hybrid materials, which are built by assembling inorganic metal ions or clusters (nodes) and polydentate organic molecules (linkers). It is their distinctive feature, the extremely large internal surface area (greater than 6000 m^2^ g^−1^) that is a direct result of the rational combination of these two building blocks. Both the node geometry and the linker connectivity can be tuned to provide the designer with fine control over pore size, surface chemistry, topology and eventually the physicochemical properties of the resulting framework^14,80^ (Fig. 2).

**Fig. 3:**
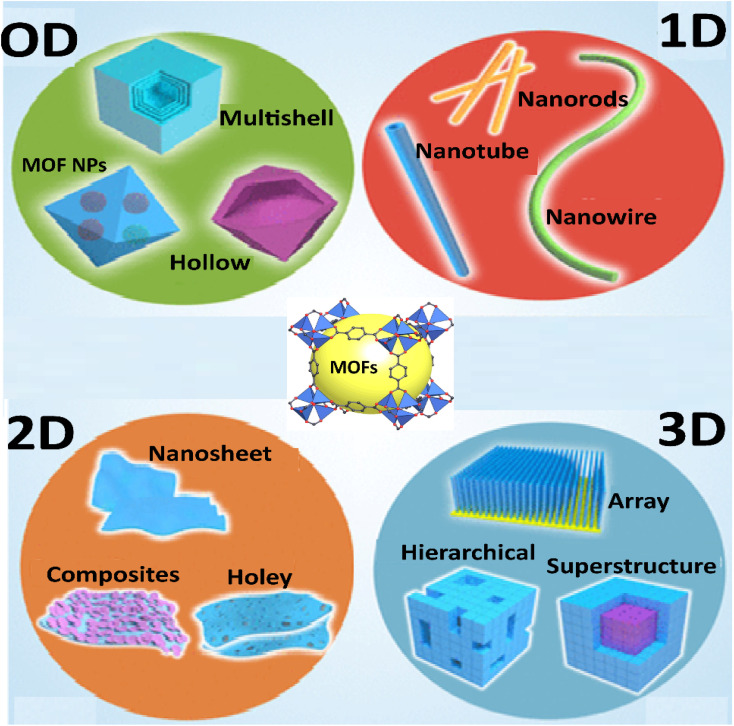
Different types showing different metal–organic frameworks, which have diverse dimensionalities and topologies. Cage-type MOFs with zero-dimensional (0D) octahedral-style structures; one-dimensional (1D) chain structures of Mo and Cr metallic ions surrounded by COOH groups and solvent DMF; two-dimensional (2D) layered frameworks in which organic linkers have at least one dimension bigger than 1 nm, including the planar or quasi-planar arrangement with different coordination modes to give rise to a wide range of structural motifs; three dimensional (3D): porous networks between dense metal–oxygen two/dimensional layers. The dimensionality is determined by the connectivity (denticity) of organo linker while its coordination geometry governs for metal node, which directly affects NLO response *via* chromophore packing density, π–π interaction strength and symmetry of crystal. Adapted from ref. 83 with permission from Elsevier, M. Tayyab, *et al.*, in *Nanomaterial-Based Metal Organic Frameworks for Single Atom Catalysis*, Elsevier, 2023, pp. 107–138, copyright 2023.

One of the key ideas in MOF design is that the node of the framework is the individual inorganic cluster or polynuclear metal complex, known as the secondary building unit (SBU). Systematic variation in the geometry of the SBU and in the length or functionality of the organic linker can be used to form structures of zero-dimensional (0D) nanoparticles up to three-dimensional extended structures. Under this section, the structural functions of both building components and the chemical variety are outlined and the combination of the two building components to form the characteristic structural attributes of the nano-MOFs are explained. The linker connectivity and SBU geometry combination generate frameworks with different dimensionalities and morphologies, and each has different property benefits. Nano-MOFs where the bulk crystal is shrunk to the nanoscale dramatically increase the surface-to-volume ratio and shorten diffusion path lengths, which are important in catalysis and sensing applications as shown in Fig. 3.^81,82^

### 4.1 Organic linkers

**Fig. 4:**
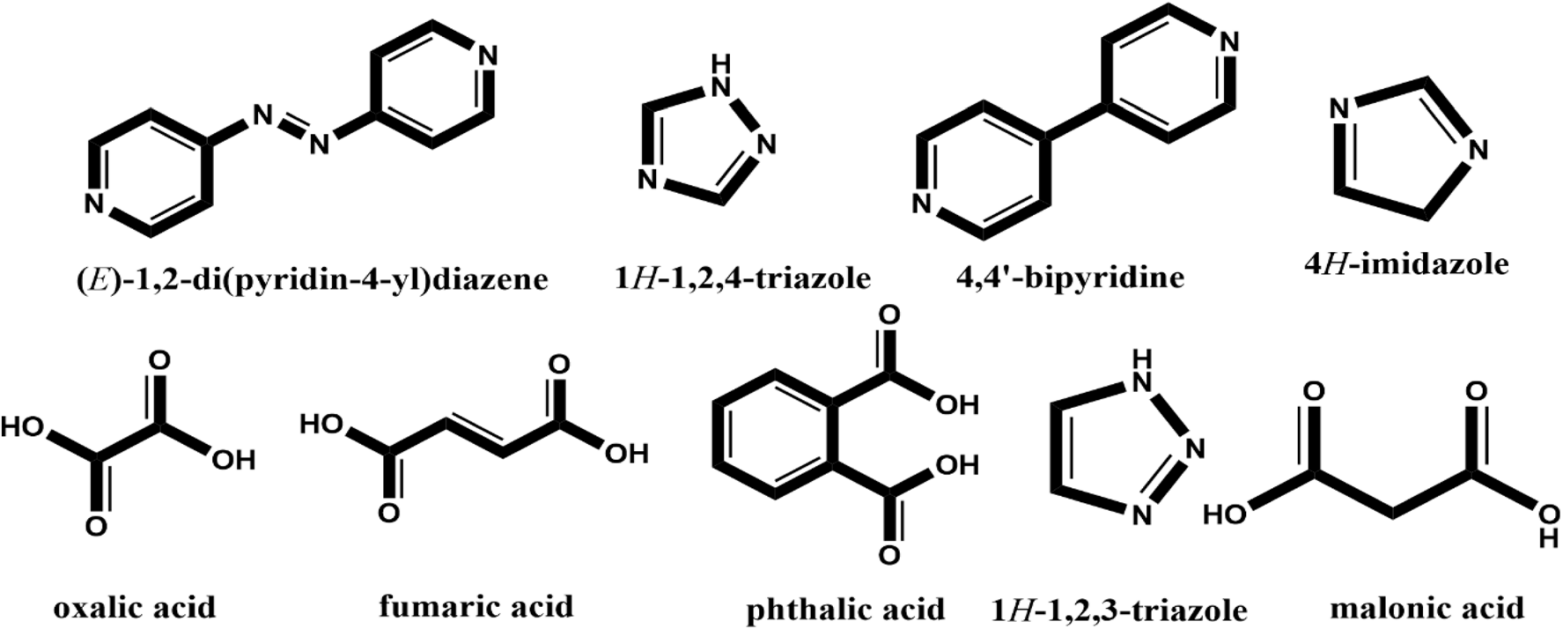
Examples of organic linkers typically used for NLO-active MOF synthesis by donor group class. First row: nitrogen-donor linkers including (*E*)-1,2-di(pyridin-4-yl)diazene, 1*H*-1,2,4-triazole, 4,4′-bipyridine, and 4*H*-imidazole. Second row: carboxylate-based linkers including oxalic acid, fumaric acid, phthalic acid, 1*H*-1,2,3-triazole, and malonic acid. Adapted with structural modifications from ref. 87; redrawn by the authors using ChemDraw.

Bridging ligands (also known as organic linkers, organic struts) are versatile molecules that bind metal nodes with coordinate bonds, forming the backbone of the porous structure. The topology, pore size, chemical affinity, and size of the linker can be determined by the geometry, length, type of donor group and functional substituents of the linker.^84,85^ The size of the pore in the final framework is directly controlled by the length of the linker. A strong method to systematically increase the size of pores is to systematically increase the length of the linker, keeping the node and topology constant. This is shown in the IRMOF series (IRMOF-1 to IRMOF-16) in which the unit cell dimension of MOF-5 is increased by various factors of 12.8 Å to greater than 28.8 Å by substituting BDC with successively longer dicarboxylates without altering the cubic topology of MOF-5.^14^ In the same way, the node branching factor and consequently topology of the framework is determined by the connectivity (denticity) of the linker: ditopic linkers prefer 1D chains or 2D sheets, tritopic linkers 3D porous networks, and tetratopic linkers prefer 2D sheets. The stiffness of the linker backbone is also a factor of importance as well. Flexible or semi-flexible linkers, in turn, synthesize breathing MOFs that can switch their structure in response to temperature, pressure, or even the presence of guests, such as MIL-53 or the [Zn(bdc)_2_(dabco)]_*n*_ system mentioned below. This structural plasticity can be used to make gated adsorption and selective molecular recognition.^14^ Topological substitution of functional groups on the linker does not change the topology but modifies the pore environment. Electron-withdrawing groups (–NO_2_, –CN) move the band gap of semiconducting MOFs to lower energy, whereas electron-donating groups (–NH_2_, –OH) move band edges upwards as a result underpinning photocatalytic design. Water adsorption is inhibited by hydrophobic groups (–CH_3_, –F) on pore walls, enhancing hydrothermal stability, which is still a significant problem in MOF application in ambient conditions (Fig. 4).^86^

#### 4.1.1 Classification by donor groups

##### 4.1.1.1 Carboxylate-based linkers

The most commonly used class in the synthesis of MOFs is carboxylate linkers. When the carboxylic acid (–COOH) groups of the carboxyl group are deprotonated, the resulting carboxylate anions interact well with metal cations, both by monodentate and bidentate bridging complexions to form strong and highly crystalline structures. The best-known linker is the benzene-1,4-dicarboxylate (BDC or terephthalate) linker, which is the backbone of MOF-5 and the MIL-53 family. Adding more carboxylate groups to the aromatic core, such as benzene-1,3,5-tricarboxylate (BTC), increases connectivity of the node, and forms frameworks with larger surface areas, such as HKUST-1 [Cu_3_(BTC)_2_].^86^ Hydroxyl-functionalised carboxylate linkers like 2,5-dihydroxyterephthalate (DOBDC) add new Lewis-basic oxygen donors, forming open-metal sites once the solvent has been removed, a major factor in gas adsorption and selective catalysis (MOF-74/CPO-27 family). Post-synthetic modification handles (such as 2-aminoterephthalate) and band-gap tuning (relevant to photocatalytic applications) is available with amino-functionalized variants.^88^

##### 4.1.1.2 Nitrogen based linkers

Imidazolates, pyrazolates, triazolates, tetrazolates, pyridines, and bipyridines are nitrogen-donor linkers that coordinate using their lone-pair nitrogen atom. The most notable of these are the zeolitic imidazolate frameworks (ZIFs): 2-methylimidazolate (2-MeIm) bridges of Zn^2+^ or Co^2+^ with a Zn–Im–Zn angle (≈145°) that resembles the SiO_2_Si angle in zeolites, forming highly stable, sodalite-topology structures including ZIF-8 ZIFs are thermally and chemically stable (much better than many carboxylate MOFs) due to the strength of the M–N coordinate bond coupled with the hydrophobic methyl substituent which protects the metal core against moisture.^32,88^

Bipyridyl and azopyridine linkers are used as pillar struts in layer-pillar designs to assemble 2D metal carboxylate sheets into 3D porous materials. The compound [Zn(bdc)_2_(dabco)]n is based on the dabco (1,4-diazabicyclo[2.2.2]octane) pillar and BDC sheets, which provides flexible structure with a pore aperture that reacts to adsorbed solvent molecules.^89^

##### 4.1.1.3 Functional linkers and mixed donor

**Fig. 5:**
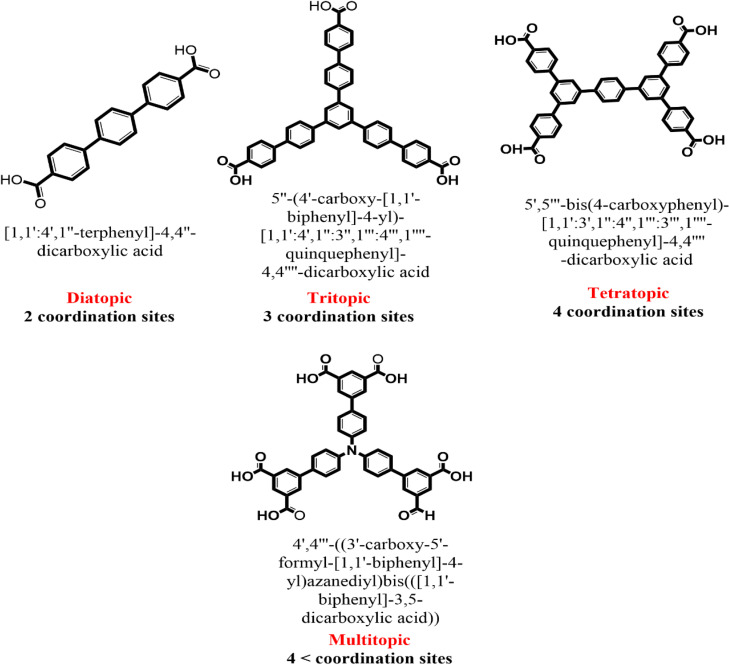
Organic linkers, also known as MOF ligands, are the connecting molecules used to connect metal nodes in the structure. An organic molecule has to have at least two coordination sites each with electronegative atoms (oxygen or nitrogen) to act as a linker. The boronic acid, carboxylic acid, phosphonic acid, sulfonic acid, amine, and heterocyclic amine are functional groups that can form coordination bonds. Using these sites, the electronegative atoms give out electron density to the metal centers, forming coordination bonds to maintain the framework. Linkers are also categorized by the number of coordinating groups – ditopic, tritopic, tetratopic or multitopic – indicating how connected each linker is to the entire network drawn by the ChemDraw on the concept reported in ref. 29 and 90.

Numerous recent designs of linkers use carboxylate and nitrogen donors as a single molecule, or add pendant functional groups (–NH_2_, –NO_2_, –OH, –SH, –F) to regulate selectivity or photocatalytic activity or post-synthetic reactivity. For example, H_2_DOBPDC (4,4′-dioxidobiphenyl-3,3′-dicarboxylate) is an elongated analogue of DOBDC that produces expanded MOF-74 analogues with larger pores and enhanced CO_2_ swing adsorption capacity. One of the most powerful design capabilities of reticular chemistry is the possibility to install virtually any organic functional group in the interior of the pore, without compromising the topology (see Fig. 5 and Table 2).

**Table 2:** Representative organic linkers used in MOF synthesis, classified by chemical class, functional groups, coordination mode, and the MOF systems they form. Abbreviations: BTC = benzene-1,3,5-tricarboxylate; BDC = benzene-1,4-dicarboxylate; DOBDC = 2,5-dioxido-1,4-benzenedicarboxylate; 2-MeIm = 2-methylimidazolate; dabco = 1,4-diazabicyclo[2.2.2]octane; NDC = naphthalene-2,6-dicarboxylate

Linker name	Chemical class	Functional groups	Coordination mode	MOF structure
BTC (benzene-1,3,5-tricarboxylate)	Tricarboxylate	3 × –COOH	Tridentate	HKUST-1
BDC (benzene-1,4-dicarboxylate)	Dicarboxylate	2 × –COOH	Bidentate	MOF-5, MIL-53
DOBDC (2,5-dihydroxyterephthalate)	Dicarboxylate	2 × –COOH, 2 × –OH	Tetradentate	MOF-74 (CPO-27)
2-MeIm (2-methylimidazolate)	Imidazolate	N–H (deprotonated)	Bridging N–M–N	ZIF-8
1,4-BDC + dabco pillar	Mixed N/O	–COOH + tertiary N	Pillar-layer	[Zn(bdc)_2_(dabco)]_*n*_
NDC (naphthalene dicarboxylate)	Dicarboxylate	2 × –COOH	Bidentate	MOF-177, IRMOF-8
4,4′-Bipyridine (bipy)	N-Donor	2 × pyridyl N	Bifunctional N	Pillared-layer MOFs
H_2_DOBPDC (dioxidobiphenyl-dicarb.)	Dicarboxylate	2 × –COOH, 2 × –OH	Tetradentate	Mg_2_(dobpdc) – CO_2_ adsorbent
BTC with –NH_2_ functionality	Amine-functionalised carboxylate	–NH_2_ + –COOH	Versatile donor	NH_2_-MIL-101, NH_2_-UiO-66
Fumarate	Dicarboxylate	2 × –COOH (*trans*)	Bidentate	MIL-88A
Malonate/propanedioate	Dicarboxylate	2 × –COOH	Bidentate chelate	Zn/Co malonate MOFs

### 4.2 Secondary building units (metal nodes)

**Fig. 6:**
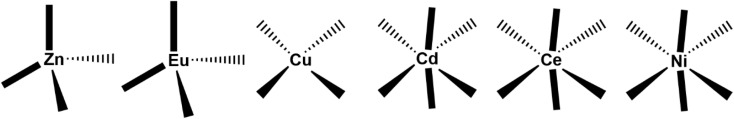
Common coordination geometries of metal nodes utilized in the construction of MOFs. Zn^2+^ within a tetrahedral node; Eu^3+^ in a nine-coordinate lanthanide node; Cu^2+^ in square planar configuration; Cd^2+^ determined to be octahedrally coordinated; Ce^3+^/Ce^4+^ are both in mixed-valence node depending on when analyzed, wherein the electron was theorized to delocalize during excitation thereby demonstrating overall molecular flexibility; and Ni, which is octahedrally coordinated. Crystal symmetry and framework dimensionality is determined by the coordination number and geometry of metal node which directly modulates NLO response through chromophore packing density and charge-transfer pathway alignment. Drawn by the ChemDraw on the concept reported in ref. 91.

The inorganic part of a MOF is the metal node or secondary building unit (SBU) which is a discrete metal ion or polynuclear metal–oxygen/metal–nitrogen cluster which serves as a branching point of the structure. The directionality of the framework and hence the topology is determined by the geometry of the SBU (the number and spatial arrangement of its coordination bonds to linkers). SBU geometry depends on three factors: (1) the coordination number of the metal ion, (2) the geometry of coordination dictated by the d-electron configuration and ionic radius of the metal, and (3) the geometry dictated by the connectivity of the bridging ligands.^91,92^ Mononuclear SBUs are single metal ions surrounded by organic linker donors, and in most instances coordinated solvent molecules which can be removed to reveal open metal sites. Common geometries are octahedral (six coordinated, *e.g.*, Cr^3+^ in MIL-101), square-planar (four coordinated, *e.g.*, Pd^2+^ in some pillared MOFs), and tetrahedral (four coordinated, *e.g.*, Zn^2+^ in MOF-5 and ZIF-8). The relatively straightforward geometry of mononuclear SBUs gives rise to less-connective nodes (2–4 points) that prefer lower-dimensional or less-robust frameworks. Metal clusters linked by oxygen or hydroxy groups are called polynuclear SBUs and they offer increased connectivity, rigidity and stability (Fig. 6).

**Fig. 7:**
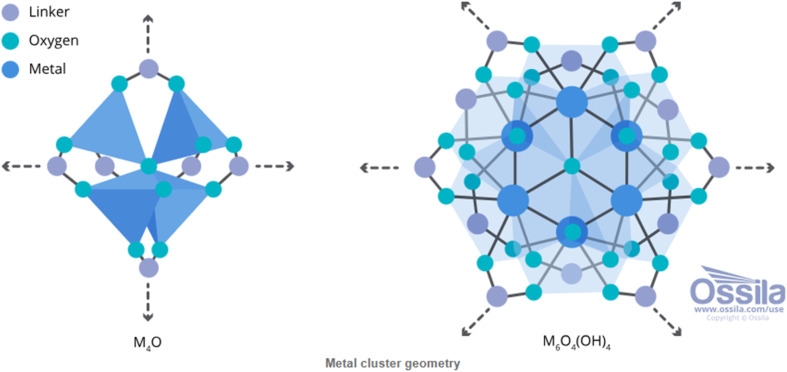
In MOF structures, the inorganic part, which is often called the metal node, metal center or inorganic center, can be a single metal ion or polynuclear metal cluster. One node can be a multilateral junction point in the extended structure and interact with multiple organic linker molecules at the same time. Assemblies composed of groups of oxygen atoms that bridge these groups of metal atoms also serve as effective linkers. Common examples of these metal–oxo clusters used in the synthesis of MOFs include M_3_O (M = Al, Fe, Cr), M_4_O (M = Zn), M_6_O_4_(OH)_4_ (M = Zr, Hf, Ce), and M_8_O_8_(OH)_4_ (M = Ti). Formally, these multinuclear assemblies are called secondary building units (SBUs). The inflexible nature of the strong bonding interactions inside each SBU determines a certain three-dimensional geometry, where the position of the linkers to be attached in a fixed spatial arrangement. This structural regularity makes sure that coordination with organic linkers is made in a regular pattern throughout the framework.^87,90,96,97^ Figure from Ossila Ltd, What Are Metal–Organic Frameworks?, https://www.ossila.com/pages/what-are-metal-organic-frameworks, accessed June 2025, copyright 2026 Ossila Ltd.

The archetypal examples are: (1) the copper paddlewheel [Cu_2_(O_2_CR)_4_], a square 4-connected SBU formed by two Cu^2+^ ions bridged by four carboxylate groups, found in HKUST-1 and many related MOFs; (2) the zinc oxide tetrahedron [Zn_4_O(O_2_CR)_6_], a 6-connected octahedral SBU that forms the node of MOF-5 and the entire IRMOF series; and (3) the zirconium oxo cluster [Zr_6_O_4_(OH)_4_(O_2_CR)_12_], a 12-connected cuboctahedral SBU that gives UiO-66 its extraordinary chemical and thermal stability.^32,93^ The thermal stability, chemical stability, and catalytic activity have far-reaching implications on the choice of metal. The high-valency metal ions (Zr^4+^, Ti^4+^, Fe^3+^, Al^3+^, Cr^3+^) form very strong metal–oxygen complexes with high charge density, which form structures of unprecedented stability – UiO-66 (Zr), MIL-101 (Cr/Fe) and MIL-53 (Al/Fe/Cr) remain crystalline weaker bonds are formed with low-valent metal ions (Zn^2+^, Cu^2+^, Co^2+^, Ni^2+^, Mn^2+^), which create structures, which can be moisture-sensitive, but highly susceptible to post-synthetic metal exchange and have rich redox and magnetic chemistry.^94^ One of the key findings that can be applicable to nano-MOF derivatization is the reduction potential threshold reported by Das *et al.* (2012):^95^ metal atoms with a reduction potential of 0.27 V and above (*e.g.*, Cu, Ni, Co) can be converted to pure metal nanoparticles after calcination in an inert atmosphere, but metals with a reduction potential of 0.27 V and below. This concept is a predictive model in the choice of MOF precursors in the designed synthesis of MOF-derived nanomaterials (Fig. 7 and Table 3).

**Table 3:** Common secondary building unit (SBU) geometries in MOFs, with representative metal ions, connectivity (number of linker attachment points), and example frameworks. SA = surface area

SBU geometry	Coordination points	Representative metals	Example MOF
Octahedral	6-Connected	Cr^3+^, Fe^3+^, Al^3+^	MIL-101, MIL-53
Triangular prism	6-Connected	Zr^4+^, Ti^4+^	UiO-66, UiO-67
Square paddlewheel	4-Connected	Cu^2+^, Zn^2+^	HKUST-1, MOF-505
Trinuclear triangle	3-Connected	Mn^2+^, Co^2+^, Ni^2+^	MOF-74, CPO-27
Cuboctahedral cluster	12-Connected	Zr^4+^	UiO-66 (Zr_6_ node)
Linear/rod SBU	2-Connected	Zn^2+^, Mg^2+^, Ca^2+^	MOF-5, IRMOF series

## 5. Computational methods and models

MOFs are coordination polymers that are porous and consist of metal ion/cluster nodes similar to metal and organic linkers, providing tunable topology, pore size and surface chemistry. They have found various uses in sensing, separation, gas storage, catalysis, and biomedicine, which rely on these structural features.^15,98–104^ DFT is now considered as the leading computational method used to elucidate the MOF structures and predict their physicochemical properties.^105,106^

Computational databases are now indexing an excess of 70 000 MOF structures, recipient of which is the Cambridge Structural Database (CSD, >900 000 structures in total), CoRE MOF database (6000 three-dimensional MOFs),^107^ and a special library of hypothetical MOFs.^108^ A combination with grand canonical Monte Carlo (GCMC) simulations^109^ and molecular dynamics (MD) has made these resources allow large-scale high-throughput screening of MOF candidates to specific applications.

### 5.1 DFT study on electronic properties of MOF and MOF-derived nanocarbons

**Fig. 8:**
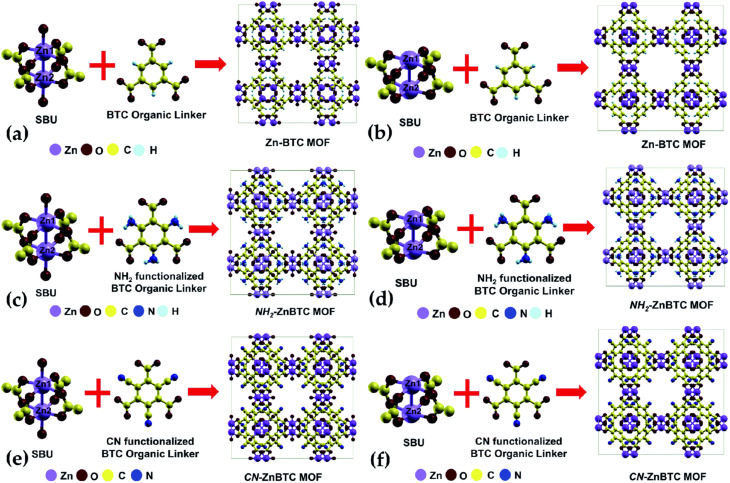
Zn-BTC MOF unit cells are made using saturated and unsaturated secondary building units (SBU) that are coordinatively saturated and linked by the benzene-1,3,5-tricarboxylate (BTC) linking group as shown with the case of pristine (a) and (b), amino-functionalized (c) and (d) and cyano-functionalized (e) and (f). Reproduced from ref. 86 with permission from the Royal Society of Chemistry, G. D. Degaga, *et al.*, *RSC Adv.*, 2019, **9**, 14260–14267, copyright 2019.

In case of carboxylate-based MOFs, HKUST-1 [Cu_3_(BTC)_2_] is the most common MOF that is subject to DFT research (see Table 4). The DFT computations done with respect to time, showed that empty Cu-3d orbital are important determinants of its electronic properties, and inter-unit (not intra-unit) excitation have to be taken considerations in order to effectively recreate the excitation spectra.^110^ In case of topological isomers TCM-8 and TCM-40, the chemical TCM-8 with the tbo topology had 43% better N_2_ uptake compared to the pto-like counterpart (76 *vs.* 53 mmol g^−1^) with the help of DFT calculations.^111^ MoF[Zn(bdc)_2_(dabco)]_*n*_ [bdc = 5 1,4-benzenedicarboxylate; dabco = 5 1,4-diazabicyclo(2.2.2)octane] was made flexible to find out the relationship between structural orientation and the molecules adsorbed by the solvent. DFT revealed that there are small energetic differences that change the orientation of DMF molecules but benzene molecules spontaneously fill in preferred sites.^89^ In the case of the Zn_3_(BTC)_2_ and its –NH_2_/–CN functionalized analogs, *ab initio* simulations with Quantum Espresso have shown that coordinatively unsaturated secondary building units (SBUs) create semiconducting MOFs whereas saturated SBUs make metallic character; cyano-groups squeeze the band gap and amino groups push band edges as shown in Fig. 8.^86^

**Table 4:** DFT-aided computational studies of MOFs and MOF-derived nanocarbons, listing key quantitative findings (electronic band gaps (eV), frontier orbital energies (eV), charge-transfer transition energies, and optical absorption onsets), DFT method and software package

MOF structures	Method of DFT/tools	Findings	Ref.
HKUST-1 [Cu_3_(BTC)_2_]	Quantum chemical calculations, time-dependent DFT, DFT+U approach with HSE06 functional	Band gap = 3.8 eV; semiconducting behavior confirmed; spin-restricted *vs.* spin-unrestricted approaches compared	110
TCM-8, TCM-40 (Cu paddlewheel)	DFT calculations	N_2_ uptake: TCM-8 = 76 mmol g^−1^*vs.* TCM-40 = 53 mmol g^−1^ (+43% increase); pore geometry is the dominant factor controlling uptake	111
[Zn(bdc)_2_(dabco)]_*n*_ (flexible MOF)	BP86/DFT/COSMO (water) protocol	Zn–ligand bond distances elongated by 0.03–0.05 Å upon guest inclusion; pore breathing mechanism validated	89
Zn_3_(BTC)_2_ + –NH_2_/–CN derivatives	Quantum Espresso plane-wave; ultra-soft pseudopotentials; PBE and HSE06 (25% HF) used	Band gap = 2.4 eV (unmodified); –NH_2_ and –CN substitutions reduce band gap by ∼0.3–0.5 eV; electronic tunability confirmed	86
M-MOF-74 (M = Mn, Co, Mg)	QTAIM, ELF, GGA functionals	Predominantly ionic M–O bonds; unusual H-bonds in Co/Mg variants; Mn shows greatest structural flexibility; CO_2_ binding energies: ∼25–45 kJ mol^−1^ across metals	112
	DFT with PBE/PW91, ultrasoft pseudopotentials, D2, QE, +U, gamma smearing		
MOF-74 analogs (hypothetical library)	Dispersion-corrected DFT + *in silico* + GCMC	61 MOF-74 analogs identified; synthesizability confirmed experimentally; pore diameters range 11–35 Å; CO_2_ uptake variation >300% across analogs	113
Cd-MOF-5	*Ab Initio* DFT; quantum mechanical studies	Bulk modulus = 13.4 GPa; band gap = 3.6 eV; cubic symmetry (*Fm*3̄*m*, space group 225); formation energy = −3.2 eV per atom	114
Zn-MOF-5	*Ab Initio* DFT; VASP	Band gap ≈ 3.4 eV; formula Zn_4_O(BDC)_3_(DMF)_*x*_, *x* = 1–2; Young's modulus ∼ 16 GPa; thermal stability up to ∼450 °C	115 and 116
MIL-88 type MOFs	DFT + GCMC simulations	MOF total energy reduced by 15–25 kJ mol^−1^ upon guest insertion; breathing amplitude: unit cell volume changes 30–40%; DFT + GCMC confirmed cooperative adsorption	117
3385 MOFs (41 topologies)	Multilevel computational + machine learning	3385 MOFs analyzed; pore-limiting diameter range: 3–30 Å; 41 distinct topologies; ML models predict mechanical stability with *R*^2^ > 0.85; bulk moduli: 0.2–50 GPa	118
MOF-901 (Ti-based, 2D)	Perdew–Burke–Ernzerhof/VASP	Reaction barrier for methyl methacrylate polymerization = 0.3 eV; band gap = 2.8 eV (visible light active); Ti–O bond length = 1.94 Å	119
6 MOF composite pairs (*e.g.*, UiO-67@MIL-88C, HKUST-1@MOF-5)	DFT (computational algorithm)	Lattice mismatch < 5% for 6 confirmed pairs; interfacial binding energies: 15–45 kJ mol^−1^; 6 experimentally verified from 90 000 candidates	120
UiO-66 + Cu-BTC/g-C_3_N_4_ composites	Nonlinear DFT (NLDFT)	Cu-BTC micropores: 5–50 nm; UiO-66: new pores formed at 0.7–1 nm; composite BET surface area up to 1200 m^2^ g^−1^; CO_2_ uptake enhanced by ∼30%	121
Zn/Cd isostructural MOFs (H_2_DMPMB linker)	Crystal14 package (DFT)	Emission shift: 441 nm (Cd) → 335 nm (Zn); Δ*E* = 0.88 eV; luminescence quantum yield: Cd = 42%, Zn = 18%; metal identity controls emission *via* MLCT	122
DUT-8(Ni) MOF	DFT (all-electron packages)	Ni–N bond length: 2.04–2.09 Å (open *vs.* closed form); gate-opening energy barrier ≈ 8 kJ mol^−1^; closed-pore phase: density = 1.12 g cm^−3^	123
M-MOF-74 (M = Zn, Ni, Co, Fe, Mg, Mn)	Polarizable force fields; Lennard-Jones; QM	Quantitative agreement across all 6 metals; CO_2_ isosteric heat of adsorption: 21–45 kJ mol^−1^; force field error *vs.* experiment < 8%; Mg-MOF-74 shows highest CO_2_ uptake	124
NiBpene flexible MOF	DFT calculations	CO_2_ adsorption enthalpy = −28 kJ mol^−1^; pore volume change upon breathing = 22%; structural transition pressure = 0.4 bar at 298 K	125
Ni-dpbz MOF {[Ni(dpbz)][Ni(CN)_4_]}_*n*_	DFT (bonding, DOS, charge analysis)	CO_2_: calculated = 204 mg g^−1^*vs.* experimental = 220 mg g^−1^ (7% deviation); electron density at Ni sites = +1.3 *e*; pore size = 6.5 × 8.2 Å	126
T-MOF-5 (H_2_BDC + ZnO)	DFT (charge transfer, DOS, binding energies)	Enhanced adsorption energy for H_2_: −14.2 kJ mol^−1^ (distorted) *vs.* −9.8 kJ mol^−1^ (ideal); net charge transfer: 0.12 *e* per molecule; CO_2_ gas loading differs by up to 25% *vs.* ideal MOF-5	127
∼8000 MOFs from CSD	MD + GCMC + Lennard-Jones	50 top MOFs identified; 110 000 MMMs evaluated; optimal pore size: 3.75–5.12 Å; porosity: 0.41–0.58; SA < 1000 m^2^ g^−1^; CO_2_/N_2_ selectivity up to 35	128
Cu-BTC (HKUST-1) and Fe-BTC	VASP (DFT)	H_2_ adsorption energy: Fe-BTC = 0.93 eV *vs.* HKUST-1 = 0.114 eV (8× difference); Fe open metal sites show stronger binding; DOS confirms d-orbital hybridization with H_2_	129
MOF-177 (Zn_4_O + benzenetribenzoate)	DFT binding energy calculations	H_2_ binding: organic linker = 2.6–3.8 kJ mol^−1^; inorganic Zn_4_O node = 2.96–4.50 kJ mol^−1^; optimal pore diameter = 11.8 Å; gravimetric H_2_ capacity = 7.5 wt% (simulated)	130
IRMOF-14, MOF-5, Ce-UiO-66, UiO-66(Zr) + catecholate	GCMC + DFT (5000 screened)	O_2_ binding energy: up to 258 kJ mol^−1^ (catecholate sites); N_2_ binding: up to 148 kJ mol^−1^; O_2_/N_2_ selectivity > 10; Ce-UiO-66 shows best performance	131
174 MOFs (acetylene separation)	GCMC simulations	Best performing SA: 600–1200 m^2^ g^−1^; porosity: 0.4–0.6; pore size: 4–11 Å; C_2_H_2_/CO_2_ selectivity up to 8.4; C_2_H_2_/CH_4_ selectivity up to 36	132
SIFSIX-3-Zn (1D channels, 3.84 Å)	DFT + GCMC simulations	Xe/Ne selectivity = 596; Xe/He = 645 (298 K, 10 kPa); Xe/Kr = 12 (273 K, 100 kPa); Xe uptake = 2.27 mmol g^−1^; isosteric heat: Xe = 28 kJ mol^−1^	133
Zr/U/Th MOF clusters	DFT+U; exchange-correlation functionals	Binding energies of UO_2_^2+^ to MOF nodes: −180 to −240 kJ mol^−1^; Zr-MOF shows highest selectivity for U over Th; coordination number = 8 for U in MOF	134
MOF-5 (water adsorption)	vdW-augmented DFT; thermodynamic integration; NEB	Energy barrier for H_2_O insertion: 0.04 eV at 300 K; 0.17 eV at 0 K; water adsorption energy = −0.65 eV; MOF-5 degradation onset at ∼15% RH	135
Cu-BTC derivatives (methyl-BTC, ethyl-BTC)	DFT-D2; classical atomistic simulations	Methyl substitution reduces BET surface area by ∼12%; water adsorption isotherm onset shifted from 20% to 45% RH; H_2_O binding energy reduced by 8 kJ mol^−1^ with alkyl groups	136
UiO-66(Zr), functionalized (–NO_2_, –NH_2_, HCl)	DFT computational simulations	Fermi energy shift: −0.8 eV (–NO_2_) to +0.5 eV (–NH_2_); Zr coordination reduced from 8 to 7; band gap tunable from 3.8 to 4.5 eV by functionalization	137
Co-MOF–S composite (Li–S battery)	DFT (binding energy per C atom)	Specific capacity = 600 mA h g^−1^; Li_2_S binding energy = −2.4 eV (*vs.* −0.9 eV for bare carbon); polysulfide diffusion barrier reduced from 0.58 to 0.21 eV	138
Zn/Co-MOF-derived Co,N-bidoped nanocarbons	DFT calculations	N-Doping level: 4.2 at%; Co–N_4_ active site binding energy for *I*_3_^−^ = −1.8 eV; power conversion efficiency: 8.3%; onset potential for catalysis = 0.12 V *vs.* RHE	139
N-Doped nanoporous carbon from ZIF (NRR)	First-principle calculations	NH_3_ yield = 3.4 × 10^−6^ mol (cm^−2^ h^−1^); N doping = 13.55%; limiting potential = −0.56 V; N_2_ adsorption energy = −0.89 eV at pyridinic-N sites	140
ZIF-67/CNT → CNT–NC–CoP	DFT studies	OER overpotential = 0.31 V; CoP active site free energy for *OH = −0.45 eV; Co–P bond weakens O binding *vs.* pure Co by 0.18 eV; specific activity = 1.8 mA cm^−2^	141
UiO-66/NH_2_-UiO-66 → C@N–C/C@N,P–C	VASP (DFT)	Built-in electric field = 0.32 V nm^−1^ at C/N–C interface; Al^3+^ diffusion barrier reduced from 0.91 to 0.34 eV; specific capacity = 148 mA h g^−1^; capacity retention: 92% after 500 cycles	142
Bimetallic ZIF-67/ZIF-8 → nanoporous carbons	DFT (pore size distribution)	Co^2+^/Zn^2+^ ratios: 0.05/0.95 → 0.67/0.33; graphitization degree increases from 12% to 74% with Co content; surface area: 900–1500 m^2^ g^−1^; pore size: 2–8 nm (900 °C, 3 h)	143
ZIF-8 → N-doped porous carbon (600 °C + NH_3_)	DFT calculations	Acetone adsorption = 417.2 mg g^−1^; N content = 13.55%; pyridinic-N binding energy = −0.72 eV; BET surface area = 1032 m^2^ g^−1^; micropore volume = 0.41 cm^3^ g^−1^	144
Zn-MOF-derived porous carbons (MOF-5, MOF-177, bioMOF-100)	DFT studies	MOF-5-derived carbon: SA = 3000 m^2^ g^−1^; MOF-177: SA = 2600 m^2^ g^−1^; bioMOF-100: SA = 4300 m^2^ g^−1^; Zn evaporation *T* > 800 °C critical for porosity generation	101

In case of DFT study of M-MOF-74 and other related compounds, investigation of M-MOF-74 (where, M = Mn, Co, Mg) with QTAIM, ELF, and GGA functionals determined a predominantly ionic character of the M–O bond in these materials, discovered unusual hydrogen bonds in Co-MOF-74 and Mg-MOF-74, and validated Lewis's acid activity at metal cation sites.^112^ Dispersion-corrected DFT in combination with *in silico* crystal assembly was also used to construct a hypothetical library of 61 MOF-74 analogues with open-metal sites having the ability to coordinate CO_2_ and Mg_2_(olsalazine) was computationally discovered as a novel analogue.^113^ For Cd-MOF-5, the initial DFT analysis showed that the bonding of Cd was mainly ionic, both the C and O bonds were covalent, the bulk modulus was 13.4 GPa, and the band gap was 3.6 eV in a cubic (*Fm*3̄*m*) structure.^114^ The Zn analogue of MOF-5 has a similar nonmetallic band gap (∼3.4 eV).^115^ The calculations of the VASP confirmed that the environment of coordinating the Zn^2+^ is not rigid, there is at least one DMF molecule coordinated in each cluster, creating a general formula of Zn_4_O(BDC)_3_(DMF)_*x*_ (*x* = 1–2) and giving an insight into the formation of MOFs and an interaction of the guest molecule.^116^

Moreover, we also observed the DFT on nanocarbon based MOFs (see Fig. 2), the hierarchical porosity and tunable morphologies, together with easy functionalization with metals, metal oxides, and heteroatoms, have led to a high level of interest in MOF-derived carbon and metal-based nanomaterials. These properties make them very useful in heterogeneous catalysis,^145,146^ electric catalysis^145^ and other electrochemical catalysis like batteries. It is worth noting that, the pyrolysis of MOFs to form nanocarbon might offer an opportunity pathway in accordance with the tenets of green chemistry.^147^ Diversity in the structures of mono and polymetallic MOFs can be used to produce a rich library of metal-containing nanocarbons with desirable properties. Unlike MOF precursors, where computational approaches have been widely utilized to analyze them, MOF-based nanocarbons have not been explored within the linear density functional theoretical approach of DFT. The literature is rather small and mostly gives attention to the electrocatalytic use. An example would be the use of cobalt- and nitrogen-doped carbon nanomaterials based on Zn/Co-containing MOFs as counter electrodes (CEs) in quantum dot-sensitized solar cells (QDSCs).^139^ The monolithic arrangement of Co and N dopants, high conductivity, large surface area, and hydrophilicity are likely sources of the high catalytic activity of these materials in the reduction of polysulfide. DFT simulations suggest that the improvement of the electrocatalytic activity is a result of the CoN_*x*_ active sites in reaction with polysulfide species. In the same manner, nitrogen-doped nanoporous carbons based on ZIF-type MOFs have been proven to be highly effective as electrocatalysts in the nitrogen reduction reaction (NRR).^140^ Since the conventional synthesis of ammonia using N_2_ and H_2_ is energy intensive, other electrochemical pathways are highly sought after. The nanocarbon reported has an NH_3_ yield of 3.4 × 10^−6^ mol cm^−2^ h^−1^ in the presence of ambient temperature of KOH where K^+^ ions tend to transfer the electron. The first-principles calculations indicate that N_2_ adsorption is known to take place on three pyridinic nitrogen atoms at the carbon vacancies which allows dissociation of N_2_ which is subsequently protonated. But iron doping prevents such active sites thus favoring the hydrogen evolution reaction (HER) against NRR, so the production of ammonia is reduced. Nanocarbons based on MOF have also demonstrated promising activity with regard to oxygen evolution reactions (OER). The following gives an example of a hybrid material formed by ZIF-67 on carbon nanotubes which is then carbonized and phosphorized in to a CoP nanoparticle embedded within N-doped carbon (CNT-NC-CoP).^141^ The material has improved OER performance since it has many active sites and better conductivity. DFT analysis blames this activity on charged CoOOH/graphene (G) interfaces. Moreover, heteroatom-doped nanocarbon of Zr-based MOFs (UiO-66 and NH_2_-UiO-66) has been explored in the case of aluminum-ion batteries.^142^ The C@N–C graded heterostructures with P variants have better conductivity and electrochemical performance. DFT simulations (in the Vienna *Ab Initio* Simulation Package) reveal progressive N and P doping to alter the electronic structure, facilitate redistribution of charges, and improve reaction kinetics, such as AlCl_4_− ion transport. In addition to electrocatalysis, graphitization and adsorption properties of MOF-derived nanocarbons have also been investigated using DFT. As an illustration, nanoporous carbons based on Zn and Co bimetallic MOFs (ZIF-8 and ZIF-67) can have their graphitization behavior tuned, with cobalt nanoparticles catalyzing graphitization when they pyrolyze at 900 °C.^143^ DFTE analysis shows that with Co content of Co_*x*_Zn_1−*n*_(MeIm)_2_ precursors, transformation of micropores to mesopores occurs, thus differentiating pore size distribution and surface properties. Moreover, ZIF-8 was pyrolyzed at 600 °C and then treated with ammonia to form nitrogen probed porous carbon that exhibits a high adsorption capacity of acetone with a capacity of 417.2 mg g^−1^ at close room temperature.^144^ DFT computations show that the large surface area, microporosity and the presence of nitrogen functional groups (pyridinic, pyrrolic, and oxidized nitrogen species) control the high adsorption capacity. Lastly, nanocarbons derived by MOF have been explored to capture CO_2_. Zn-based MOFs (MOF-5, MOF-177, bioMOF-100) are subjected to pyrolysis to generate porous carbon materials that can be used to sequester CO_2_ even in moist environments.^101^ According to the DFT studies, relative distribution of the micro and mesopores through the carbon framework affects the adsorption performance.

### 5.2 In stability and mechanical properties of MOFs

Some MOFs are not very stable and this may limit their use and application on a large scale. Hence, enhancing MOF stability is an essential area of research interest, especially in light of their highly porous nature and chemical adaptability.^148^ Their instability when exposed to moisture or water is one of the greatest challenges as it drastically interferes with their handling and usage. Notwithstanding this issue, many water-stable MOFs have been prepared including those frameworks that can be maintained in a broad pH range, *e.g.*, stable in highly acidic conditions (*e.g.*, AlTCS-1), which can even be used in aqua regia) to very basic conditions (*e.g.*, PCN-601). Thus, chemical stability should not be considered an insurmountable barrier. Rather, computational chemistry gives useful ideas on how to increase the stability of MOFs. An example of this is seen in the synthesis of MIL-88-type MOFs,^117^ which has been shown to incorporate 2,4,6-tri(4-pyridyl)-1,3,5-triazine (tpt) and [Co_2_(ina_3_(H_2_O)_2_]^+^ where, (ina = isonicotinate) in the synthesis of the system, reducing the energy of the these findings were validated by DFT calculations and Grand Canonical Monte Carlo (GCMC) simulation which showed enhanced stability in aqueous conditions in accord with experimental observation. The mechanical properties are key in establishing robustness and applicability of MOFs in the industry. The computational study is performed at a multilevel that has allowed the creation of an interactive map, which correlates mechanical properties with structural features.^118^ Bulk and shear moduli among other properties are highly dependent on factors like topology of the framework, pore size, node type, coordination environment and the geometry as a whole. A systematic classification of ligands and metal nodes was performed in a detailed study of about 3400 MOFs in more than 40 different network topologies in terms of their length and coordination number, respectively. The resulting computational model enables predicting the mechanical properties automatically, which enables the identification of MOFs with improved mechanical stability that can be used in high-pressure industrial processes. The mechanical stability is also affected by guest molecules. Indicatively, the DFT calculations with the Perdew Burke Ernzerh of (PBE) functional, in the Vienna *Ab Initio* Simulation Package (VASP), were applied to explore the hypothetical titanium-based MOF-901.^119^ It was demonstrated that the use of methanol (Me–OH) adsorption stabilizes the two-dimensional framework in the presence of the guest molecules and without them. Additional computational studies showed that ethyl 1,2-bromophenylacetate dehalogenation had a Ti(iv) → Ti(iii) transition, which took place without an activation barrier, and also a low-energy barrier (∼0.3 eV) methyl methacrylate polymerization initiated by ethyl phenylacetate radicals. Also, the mechanical and structural characteristics of other Zn–(zinc), Zr–(zirconium), and Cu-MOFs, such as IRMOF-10, MOF-5, HKUST-1, IRMOF-14, and the UiO-66/67, have been successfully explained with the help of ab initio-based force fields, again demonstrating the power of computational chemistry in predicting and optimizing MOF performance.

### 5.3 Some new frontiers in DFT study of MOFs (2018–2026)

The recent literature has greatly contributed to DFT applications in MOF science in four new directions, namely: (1) high-throughput screening of linkers with hybrid DFT-machine learning; (2) orbital engineering of photocatalytic MOFs; (3) electronic structure of actinide-containing frameworks; and (4) periodic DFT to optimize hydrogen storage. The seven main studies that have been summarized under this section:

Achour *et al.* (2026) used the protocol B3LYP/6-31G(d,p) DFT to calculate HOMO–LUMO gaps of 195 naphthalene diimide (NDI) derivatives – which are potential MOF linkers. Their hybrid DFT-LightGBM machine learning scheme attained a test *R*^2^ of 0.86 and RMSE of 0.25 eV in a range of gaps of 1.13–3.63 eV, and provided a 10^6^-fold acceleration per molecule. Across the NDI scaffold, SHAP analysis identified electro topological E-state indices as the dominant features governing gap modulation, consistent with substituent-driven π-polarization. This is a radical change to a case-by-case optimization of DFT to population-wide pre-screening of linkers.^149^

Li *et al.* (2018) systematically studied the functionalization of MIL-125 (Ti-MOF) with –NH_2_, –OH, and –NO_2_ with GGA-PBE and HSE06. A key result was that the division of labor between organic and inorganic components, the VBM (valence band minimum) is solely controlled by the organic linker, and the Ti–oxo node is solely controlled by the conduction band minimum (CBM). The NH_2_ functionalization of the VBM decreases the band gap between pristine and tri-functionalized (NH_2_)_3_–MIL-125 by narrowing the 4.08 eV to 1.48 eV band gap, and moving the absorption to the visible and infrared spectrums at high frequencies as a result of N lone pair donation.^150,151^ Pandey *et al.* (2020) performed GGA-PBE, DFT+U, HSE06, and B3LYP calculations on MOFs based on Zr-, Th-, and U-based on the same Mx cluster SBU geometry. Zr- and Th-MOFs were identified as ligand-to-metal charge transfer (LMCT) insulators having band gaps of about 3.50 eV and HOMO based on O(2p) and LUMO based on metal d/f states. Another important result was that U-MOF appears to be mistakenly predicted as a metallic material without DFT+U corrections; U(5f) localization of electrons makes it a Mott insulator with a band gap of 2.38–2.70 eV. The spin–orbit coupling was negligible, and hybrid functionals overcame gaps by as much as 57 percent compared to experiment (∼3.30 eV).^152^

Elmi Fard *et al.* (2024) surveyed the applications of DFT in *Z*-schemes heterojunctions MOFs in photocatalytic wastewater treatment and water splitting. Analysis of band structure of Bi_2_WO_6_/Bi-MOF showed opposite gap characters whereby, the direct band gap of Bi-MOF is 2.59 eV, whereas the indirect band gap of Bi-MOF is 1.88 eV, with Bi 6s and O 2p orbitals prevailing in the valence band. The reactive sites of pollutant molecules (TC-HCl, SMX) were identified by HOMO–LUMO and electrostatic potential (ESP) mapping, which showed the presence of nucleophilic, electrophilic and radical attack vulnerability areas. The reported efficiencies of degradation were 9399% across the systems reviewed with band gaps of 1.45–3.53 eV.^153^ In order to justify the selective adsorption of Congo Red by a La-MOF–NH_2_–Fe_3_O_4_ magnetic adsorbent, Mirzaee Valadi *et al.* (2020) used B3LYP/6-31G(d) DFT to compute global reactivity descriptors chemical hardness (*η*) and electronic chemical potential (*µ*) and electrophilicity index. The lowest chemical hardness was observed with Congo Red (*η* = 1.094 eV) which is the most reactive molecule of all dyes used. Extreme values of −146.31 and −29.56 kJ mol^−1^ were found on the sulfonate groups of the Congo Red (localization of HOMO orbitals) which accounts for its rapid and specific electrostatic adsorption (92.02% removal in 2 min; *q*_max_ = 716.2 mg g^−1^).^154^

Prasetyo and Pambudi (2021) calculated periodic DFT calculations with the PBESol functional and the D3 dispersion correction (CP2K 6.1) of the fluorinated MOF-801 (a Zr-MOF). Lattice parameter (*a* = 1.788 nm, 0.28% deviation) was well reproduced by the computational approach. Replacement of –OH groups by –F atoms in fluorination formed Zr–F binding sites (bond distance 0.225 nm) that enhanced H_2_ binding energy of the material by changing a value of 3 kcal mol^−1^ (pristine) to 5 kcal mol^−1^ (fluorinated) per mole. Notably, the charge analysis using DDEC6 revealed that the partial charge of Zr^4+^ did not change (approximately 2.34 *e*), indicating that the enhanced binding is caused by the high-electronegativity-positron polarization of F and charge transfer and not charge redistribution at the metal node. The main H_2_ adsorption position changed to the electronegative site of Zr–F.^155^ DFT has already become an essential workhorse in all areas of MOF science, including the determination of electronic structures and the prediction of mechanical properties, the screening of gas separations and catalyst designs. DFT combined with GCMC, MD, machine learning, and high-throughput database screening has made it possible to computationally characterize tens of thousands of MOF structures, decreasing the amount of effort required in experiments many times over.

Recent studies have reportedautomated prediction of mechanical stability of 3385 MOFs;^118^ 50 best performing MOFs to CO_2_/N_2_ membrane separation of a database of approximately 110 000 MMMs;^76^ prediction and experimentally validated 6 crystallographically compatible MOF pairs^120^ the creation of polarizable force fields at open metal sites for CO_2_ adsorption.^124^

The seven recent papers (2018–2026) point out some new frontiers: (1) hybrid DFT-ML can now screen linker libraries 10^6×^ times faster with *R*^2^ = 0.86 accuracy; (2) orbital rehybridization with metal doping can be used to reduce band gaps and achieve >94% catalytic selectivity; (3) actinide (U 5f) states in MOFs can only be treated correctly by DFT+U; (4) periodic DFT plus dispersion corrections gives reliable predictions of H_2_ binding enhancement using strategic fluorination, and (5) reactivity indices (chemical hardness, ESP mapping) calculated by DFT reliably predict adsorption selectivity among competing molecular targets.

**Fig. 9:**
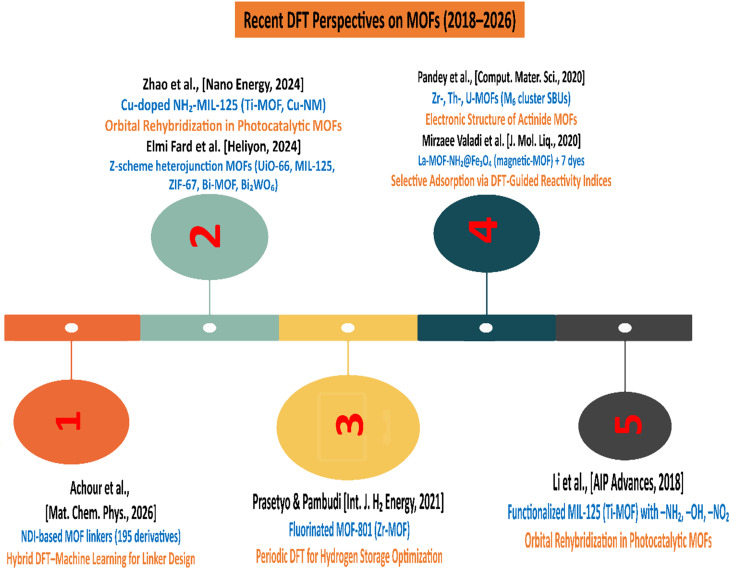
The graphical representation of the DFT perspective on MOFs.

In the case of MOF-derived nanocarbons, there are still no systematic and thorough DFT studies focused on electrocatalysis (NRR, OER, HER) and battery applications.^101,139–144^ There is much potential in the computational characterization of nanocarbon structures, pore engineering and surface chemistry, where DFT techniques that are well developed on MOF precursors have not been transferred fully (Fig. 9).

### 5.4 NLO across MOF families

#### 5.4.1 Zr-Based MOFs

MOFs belonging to the family of Zr-based UiO-66 (UiO = Universitetet i Oslo) are some of the most thermally and chemically stable MOFs available, and therefore, make good NLO candidates that need materials stability when hit by laser. Parent UiO-66 is a topology of 1,4-benzenedicarboxylate (BDC) linkers between the Zr_6_O_4_(OH)_4_ octahedral clusters in a face-centered cubic topology. Since the parent UiO-66 is centrosymmetric, the bulk SHG response disappears; however, functionalization of the BDC linker with electron-withdrawing and electron-donating groups systematically alters the electronic structure and can break centrosymmetry at the molecular scale, thereby improving hyperpolarizability. The first fully hybrid DFT (HSE06) study of the optical properties of UiO-66 and its analogues with mono and bifunctional push–pull BDC linkers was performed by Schneider *et al.*^156^ This shift of the band gap between the pristine UiO-66 (approximately 3.9 eV) and UiO-66–NH_2_ (approximately 2.8 eV) by approximately 1.1 eV occurred with the addition of an amino group (electron donor), as expected in the experimental data. A novel push–pull UiO-66–(NH_2_,NO_2_) with an amino donor on one end of the BDC linker and a nitro acceptor on the other end demonstrated a band gap of 2.78 eV with a refractive index tunability range of between *n* = 1.37 to *n* = 1.78 in the series and comparable to high-performance optical glasses. This conceptually similar push–pull BDC design to the D–A linker design that we have used in our molecular chromophores in which *N*,*N*-dimethylamine acceptors are paired with NO_2_ or CN acceptors have had a consistent and large *β*_0_ effect.^40,157^ A model of the linker field calculated by DFT by Diamond *et al.* showed that BDC linker functionalization in UiO-66 can alter the valence band energies by up to 2 eV with a mixture of σ-donation and π-donation/acceptance. It gives a design rule of DFT directly relevant to NLO engineering: electron-donating groups on the BDC linker raise the HOMO, and electron-withdrawing groups stabilize the LUMO, which work together to reduce the band gap and increase optical polarizability.^158^ Zhestkij *et al.* also experimentally showed that UiO-66 microcrystals have a third-harmonic generation (THG) at 450 600 nm when irradiated with a strong laser pulse, with a saturable absorption coefficient of *β*_eff_ = −1.415 × 10^−2^ cm GW^−1^ at 515 nm, confirming the NLO capability.^159^

#### 5.4.2 Ti-Based MOFs

**Fig. 10:**
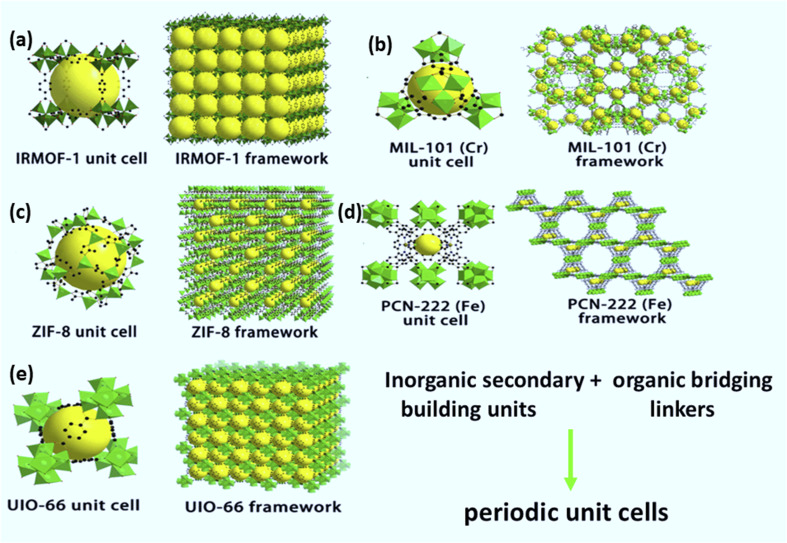
Examples of five typical MOF structures emphasizing the connection to inorganic secondary building units, organic bridging linkers, periodic unit cells and extended 3D structures. The examples of IRMOF-1 (a), ZIF-8 (b), MIL-101(Cr) (c), PCN-222(Fe) (d), and UiO-66 (e) are different families of MOFs with different topologies, metal nodes, and pore structure. Drawn by the authors based on the concept reported in ref. 161.

Another NLO MOF family that has been extensively studied using DFT is titanium-based MIL-125. The photocatalytic activity of this kind of ligand can be attributed to a ligand-to-metal charge transfer (LMCT) process: when light is absorbed, an electron is lost to the Ti_8_O_8_(OH)_12_ cluster by a BDC linker, similar to the D → A charge transfer pathway that we have described in molecular D-80CA systems using TDM and NPA analyses.^160^ Functionalization of the BDC linker of MIL-125 with electron-donating amino groups (to form MIL-125–NH_2_) with DFT calculations revealed a bathochromic shift in the absorption onset and a decrease in optical band gap of MIL-125 (3.5 eV) to 2.4 eV, allowing visible-light harvesting.^48^ The similar MIL-126 and MIL-127 constructions with longer BDC linkers even more strongly increase SHG intensity, showing the importance of π-conjugation extension as displayed in Fig. 10.

#### 5.4.3 Cu-Based MOFs

Copper-based conductive MOFs, especially Cu-HHTP (HHTP = 2,3,6,7,10,11-hexahydroxytriphenylene) are a special type of NLO-active MOFs in which charge delocalization is no longer localized in isolated linker molecules, but by the 2D π-conjugated metal–organic structure. Liu *et al.* produced controlled growth orientations (Cu-HHTP [001] and [100]) of the films and proved through DFT calculations that anisotropy resulted in 3rd nonlinear susceptibility of Cu-HHTP [001] (*γ* = 2.36 × 10^−3^ esu) being 9-fold higher than that of Cu-HHTP [100] (*γ* = 2.60 × 10^−31^ esu), due to the anisotropic charge transfer along the [001] direction. More importantly, the density of states calculated by DFT showed that the [001] orientation allows a more efficient charge transport across the metal-HHTP network, resulting in a higher NLO third-order activity. The effect of an external electric field on enhancing the NLO response was further enhanced by using an external electric field: *γ* increased to 3.84 × 10^−5^ m W^−1^ at10 V, compared to 7.60 × 10^−6^ m W^−1^ at 0 V with Cu-HHTP [001].^48^ Zhang *et al.* prepared Cu-bpy (bipyridyl-linked Cu(i) MOF) and did first-principles DFT calculations with a band gap of 2.39 eV, which is in line with the experimental band gap of 2.28 eV.^65^ Cu-bpy demonstrated reverse saturable absorption (RSA) with *β* = 100 cm GW^−1^ and an optical limiting threshold of 0.75 J cm^−1^, the lowest ever reported as a MOF-based NLO material and in direct analogy to optical limiting applications of NLO chromophores. The energy band diagram calculated using DFT validated that the photon energy of the 532 nm, excitation laser (2.33 eV) matches the band gap (2.28 eV) and thus the 532 nm excitation laser (2.33 eV) allows single-photon absorption (1PA) to occur as the governing NLO process.

#### 5.4.4 Bismuth-based MOFs

Recently, Wang *et al.* reported a bismuth-based organic framework (Bi-NBC) with a triphenylamine (TPA) carboxylate ligand (the same TPA donor unit we have widely used in our D–π–A and D–D–A chromophore designs). And it showed to have very large second-harmonic-generation (SHG) response due to its non-centrosymmetric (NCS) crystal structure.^162^ In the DFT calculations, the large potential difference between the Bi^3+^ metal ions create a high local electric field, which greatly enhances charge separation and charge carrier transport in the photocatalysis. In Bi-NBC's, SHG is stronger than that of many traditional NLO crystals, paving the way for new bismuth-based MOFs. The combination of the NLO-active TPA chromophore-based linker and the non-centrosymmetric arrangement of the Bi^3+^ coordination sites represent a perfect fit of our molecular NLO design and MOF crystal engineering.

#### 5.4.5 Bimetallic MOFs

A relatively new strategy in MOF-NLO is the design of bimetallic frameworks, where the strain field generated by two different metal ions boosts polarization asymmetry and thus SHG. Hu *et al.* designed a series of Zn/Cu bimetallic MOFs (MOF1-MOF9) with different ratios of metal ions, and investigated their SHG properties at near-infrared wavelengths. MOF3 with an optimal Zn : Cu ratio showed a record-breaking SHG enhancement (*d*_33_ ≈ 19.86 pm V^−1^), which is about 10 times larger than that of pristine Zn-MOF (1.65 pm V^−1^) and Cu-MOF (3.88 pm V^−1^), and many commercially available NLO crystals. The local centrosymmetry breaking and polarization asymmetry induced by the strain field at the Zn/Cu interface were confirmed by DFT calculations, and the SHG enhancement was attributed to the breaking of local centrosymmetry and the introduction of polarization asymmetry.^163^

#### 5.4.6 Lanthanide MOFs

**Fig. 11:**
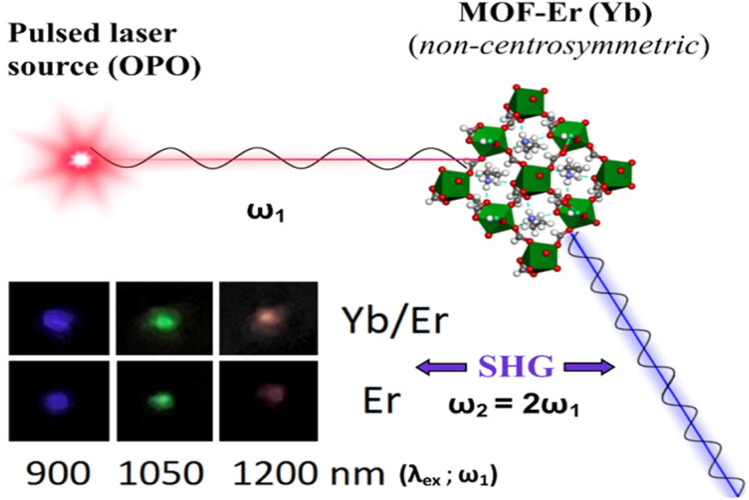
Demonstration of SHG process in non-centrosymmetric lanthanide-based MOFs. The inset (lower left) shows the digital photographs of MOF-Yb^3+^/Er^3+^ (upper row) and MOF-Er^3+^ (lower row) under the excitation of pulsed laser at 900, 1050 and 1200 nm, showing the visible emission through the frequency doubling process. Adapted from ref. 76 with permission from the American Chemical Society, M. Runowski, *et al.*, *ACS Appl. Mater. Interfaces*, 2023, **15**, 3244–3252, https://doi.org/10.1021/acsami.2c22571, copyright 2023 American Chemical Society.

**Fig. 12:**
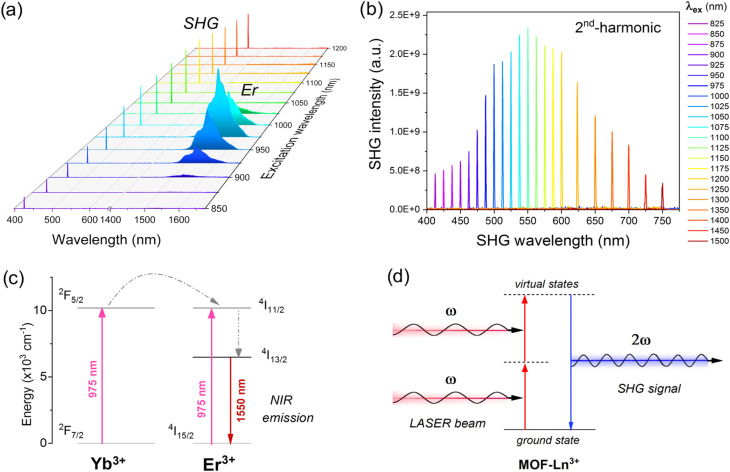
Spectroscopic data and photophysical analysis of the MOF-Yb^3+^/Er^3+^ compound (2). (a) Emission spectra collected under a series of excitation wavelengths (*λ*_ex_ = 850–1200 nm), displaying the concurrent SHG response and the characteristic Er^3+^ near-infrared luminescence centered at approximately 1550 nm. (b) Superimposed SHG spectra of the same material spanning an extended excitation range (*λ*_ex_ = 825–1500 nm), demonstrating the broadband tunability of the SHG signal. (c) Schematic energy level diagram describing the radiative and nonradiative pathways involved in Yb^3+^ to Er^3+^ energy transfer, with emphasis on the NIR emitting transition of Er^3+^. (d) Energy level diagram illustrating the physical basis of second-harmonic generation within the lanthanide-based MOF framework. Reproduced from ref. 76 with permission from the American Chemical Society, M. Runowski, *et al.*, *ACS Appl. Mater. Interfaces*, 2023, **15**, 3244–3252, https://doi.org/10.1021/acsami.2c22571, copyright 2023 American Chemical Society.

The lanthanides metal–organic frameworks have the advantage of the spectroscopic characteristics of 4f-electron metal center such as typical NIR luminescence and high spin–orbit coupling. The majority of Ln-MOFs are centrosymmetric and, therefore, do not exhibit SHG. Non-centrosymmetric Ln-MOFs made with achiral organic substrates in a single synthetic step, which allow Ln to exhibit strong and tunable SHG in the NIR range (800–1500 nm), however, were shown to be possible with careful topology selection.^164^ The co-doped MOF-Yb^3+/^Er^3+^ and isostructural MOF-Er^3+^ systems were found to have high SHG activity when the NIR luminescence of Er^3+^ at 1550 nm was detected, creating a combined NLO luminescent thermometry platform as shown in Fig. 11 and 12.

#### 5.4.7 Porphyrin-based 2D MOFs

Two-dimensional porphyrin-based MOFs are a combination of the extremely large hyperpolarizabilities of metalloporphyrin chromophores with the spatial organization that the MOF architecture can provide. Lin *et al.* prepared oriented 2D ZnTPyP(Co) thin films in which ZnTPyP is a zinc tetrapyridylporphyrin containing cobalt ions coordinated to the pyridyl rings in which a layer-by-layer method and observed a third-order nonlinear susceptibility of *χ*^(3)^ ≈ 2.63 × 10^−7^ esu.^70^ DFT and experimental Z-scan results verified that when the cobalt in the porphyrinic core is replaced, delocalization increases due to metal d-orbital involvement, and *χ*^(3)^ is greater than zinc-only analog. The nanosheet array which is vertically oriented maximizes the effective optical path length further enhancing the third-order NLO response. The architecture is an example of how MOF architecture can be used to systematically optimize the performance of NLOs by harvesting the combined benefits of chromophore selection (porphyrin), metal node selection (Co *vs.* Zn), and geometric orientation (vertical alignment).

#### 5.4.8 Zeolitic imidazolate framework (ZIF-8)

Zeolitic imidazolate frameworks (ZIFs) – and in particular ZIF-8 – built by using Zn^2+^ as nodes and 2-methylimidazolate as linkers have gained interest as NLO materials because of their high chemical stability, and they can be prepared as thin films and polymer composites. Van Cleuvenbergen *et al.* showed that ZIF-8 has a high SHG signal, on par with the well-known inorganic crystal KH_2_PO_4_ (KDP), even though it is synthesized using achiral building blocks. This result is supported by DFT calculations of the non-centrosymmetric *I*4̄3*m* space group of ZIF-8 and it demonstrates that relatively simple linkers such as imidazolates can readily generate significant SHG when assembled into a non-centrosymmetric MOF structure.^165^ Mezenov *et al.* also applied ZIF-8 by using it to prepare composite NLO materials with optical responses that can be tuned by incorporating it into polymer matrices.^165^

#### 5.4.9 NLO response in TPA-based MOFs

The TPA-based metal–organic structures (MOFs) have come to be significant category of nonlinear optical (NLO) materials, with robust electron donating capability, a multi-branched 5-conjugated framework, as well as have a tendency to form effective donor–π–acceptor (D → A) complexes, in crystalline arrangements.^166–168^ Implantation of TPA-based polycarboxylate ligands into MOFs has been discovered to be a good approach to improve the unidirectional charge transfer intramolecularly (ICT), which is a decisive factor to control the nonlinear optical response. In this regard, Yan-Ping He *et al.* have emphasized that TPA-based ligands, especially tri-, tetra-, and hexacarboxylates allow the formation of highly conjugated structures that have better optical properties such as the second-order nonlinear optical activity, as electron delocalization and structural programming can be improved.^169^ Recent research has shown that, when crystallizing in non-centrosymmetric structures TPA-based MOFs can exhibit a high second-harmonic generation (SHG). As an example, Longfei Lei *et al.* have described a MOF (Bi-NBC) based on a triphenylamine-based ligand, whose non-centrosymmetric structure and high internal electric field formed around Bi centers evoked an intense SHG signal.^162^ The authors placed great importance on the fact that heavy metal nodes and electron rich TPA ligands work together successfully to enhance charge separation and increase the second-order nonlinear susceptibility. Equally, researchers investigating lanthanide-based MOFs by Yating Wan *et al.* found that crystal symmetry and the character of the metal ion were key determinants of SHG activity with non-centrosymmetric Ln-MOFs reacting with a strong nonlinear optical response.^170^ Besides intrinsic effects of ligands, structural engineering of MOFs are also important to control NLO activity. Zhihui Chen *et al.* also showed that in structure change of a 7-fold to an 8-fold interpenetrated MOF, a significant 125-fold improvement in the intensity of SHG was observed, which was explained by more intensive π–π contacts and high oscillator strength in the framework.^171^ Similarly, Menglong Zhu *et al.* also found that strain engineering in bimetallic Zn/Cu-MOFs can lead to a significant increase in the second-order nonlinear susceptibility, with the best systems having *χ*^2^ values (∼19.86 pm V^−1^) multiple times higher than in their monometallic counterparts.^163^ They improved as induced structural anisotropy and enhanced electronic coupling in the strained state. Abazari *et al.* found a nonlinear absorption coefficients (*β*) so (2.92–14.27) × 10^−3^ cm W^−1^ and nonlinear refractive indices (*n*_2_) of (6.6–19.1) × 10^−8^ cm^2^ W^−1^ in a tablet-layered Co-MOF with mixed ligands they characterized by non-linear absorption and non-linear refraction index. A mixed-ligand Zn(ii)-MOF (NH_2_–Zn-MUM-6) integrated a high nonlinear refractive index (30.83 × 10^−8^ cm^2^ W^−1^) and *β* value of 4.06 × 10^−4^ cm W^−1^, thereby linked to an extended 3-dimensional 8 cm W^−1^ independent association between aromatic ligands and Zn centers [Zn-MOF and ZSTU-10] showed a high level of two-photon absorption and self-defocusing, which suggests that the interpenetrated structure can be used as an optical limiting device and photonic modulation device.^172^

Donor–acceptor interactions have also been studied to affect nonlinear response in TPA-based covalent organic frameworks (COFs), which offer an understanding of the behavior of related MOF systems. According to Jianhong Jia *et al.*, TPA-based COFs have been found to demonstrate saturable absorption with nonlinearity absorption coefficient of between −1.23 to −2.02 × 10^−9^ m W^−1^, where an increased donor–acceptor interaction and longer electron transport chains led to a high nonlinear performance.^173^ These results also enable the significance of D–π–A design approaches in maximizing NLO characteristics of TPA-based schemes.

Besides ligand and metal effects, external modulation strategies have been demonstrated to have substantial improvements on NLO properties in MOFs. To illustrate, Babusenan *et al.* showed that cavity-controlled improved SHG in MOF microplates can prompt a resultant nearly an order of magnitude rise in SHG strength owing to resonance impacts.^174^ Likewise, the usefulness of investigation in two-dimensional (2D) layered materials has demonstrated that the decreased dimensionality and augmented interaction between light and matter can remarkably enhance SHG efficiency, creating potential future avenue of creating 2D TPA-based MOFs to a path of the attainment of best nonlinear optical performance.^175^

In general, molecular design and framework engineering provide a complex control of the nonlinear optical activity of triphenylamine-based MOFs. Some of these important factors are strong donor–acceptor interactions, long π-conjugation, metal–ligand charge transfer, and the relatedness of the framework, and structure, including interpenetration and dimensionality. TPA units have been shown to greatly increase intramolecular charge transfer and polarizability, and more complex techniques like strain engineering, cavity modulation, and dimensional reduction can increase the nonlinear optical response even faster. The combination of these strategies makes TPA based MOFs, great potential candidates to the next generation photonic devices, such as optical limiting, optical switching, optical frequency conversion, and integrated optoelectronic devices.

##### 5.4.9.1 Structural features driving NLO activity in TPA-MOFs

Three co-operative structural features render the triphenylamine core an UHF efficient NLO building block. First, the propeller arrangement of three phenyl arms affords a π-conjugation network that is three-dimensional (3D) able to sustain multidirectional ICT and gives rise to octupolar rather than dipolar charge-transfer character and it has been shown that geometries capable of sustaining this type of ICT result in larger two-photon absorption cross-sections than those typically associated with linear dipolar chromophores of equivalent molecular weight.^6^ Second, the coordination of the peripheral carboxylate or pyridyl arms to metal nodes further locks the propeller conformation thereby quelling non-radiative decay pathways and compounding the optical response. Third, since the nitrogen lone pair acts as a powerful electron donor in D–π–A architectures, a large Δ*µ* (Δ*µ* is the ground-to-excited-state dipole moment change) directly increases *β* through the two-state model: *β* ∝ Δ*µ* × *f*/Δ*E*^3^ (where *f* is the oscillator strength and Δ*E* is the CT transition energy).^176^

##### 5.4.9.2 Quantitative NLO performance of TPA-based MOFs

Quantitative NLO parameters for known TPA-based MOFs and benchmark values are compiled in Table 4. Bi-NBC, the bismuth-organic framework derived from the triphenylamine-based tricarboxylate linker NBC [4′,4‴,4‴″-nitrilotris([1,1′-biphenyl]-4-carboxylic acid)], crystallizes in non-centrosymmetric space group and exhibits a huge SHG response with at least three times intensity as that of KDP due to cooperative action from electron-rich TPA nitrogen donor core as well as large internal electric field induced by Bi^3+^ coordination sites.^162^ The two TPA-derived MOFs tPPD-Zn and tPPD-Cd are constructed from the tetraphenyl phenylenediamine chromophore linker H_4_TPBD coordinated with metal centers Zn^2+^ or Cd^2+^ [Fig. 3] which demonstrate that chromophore packing density directly controls two-photon absorption (TPA) efficiency: by generating nine times larger 2PA action cross-section of tPPD-Cd than tPPD-Zn through higher interpenetration-induced chromophore density (intermolecular spacing reduced from 15.1 Å to 11.5 Å).^177^ The Cu-bpy MOF shows a nonlinear absorption coefficient of 100 cm GW^−1^ and an excellent OL threshold of only 0.75 cm^2^ at sub nanosecond excitation (532 nm) which is an attractive combination for optical limiting applications which constitutes one of the lowest thresholds ever reported for MOF-based optical limiters (Table 5).^65^

**Table 5:** The NLO parameters of TPA-based MOFs

MOF	TPA linker	Metal	NLO type	Key parameter	Technique	References
Bi-NBC	NBC (TPA-triphenyl carboxylate)	Bi^3+^	SHG (2nd order)	SHG ≫ KDP; non-centrosymmetric	Kurtz-Perry powder	162
tPPD-Cd	H_4_TPBD (TPA-derived D–π–A)	Cd^2+^	2PA (3rd order)	*σ*_2_ ∼ 9× that of tPPD-Zn; spacing 11.5 Å	fs Z-scan	177
tPPD-Zn	H_4_TPBD (TPA-derived D–π–A)	Zn^2+^	2PA (3rd order)	*σ*_2_ baseline; spacing 15.1 Å	fs Z-scan	177
Cu-bpy	4,4′-bipyridyl (N-donor)	Cu^+^	OL (3rd order)	*β* = 100 cm GW^−1^; OL = 0.75 J cm^−2^	ns Z-scan 532 nm	65

##### 5.4.9.3 External modulation of NLO response in TPA-MOFs

The defining advantage of porous MOFs over molecular chromophores, namely their ability to be externally modulated to control the NLO response. The three factors are: (1) electric field tuning: 10 V applied to Cu-HHTP films raised *β* from 7.60 × 10^−6^ m W^−1^ → 3.84 × 10^−5^ m W^−1^ which a factor of five improvement, with DFT redistribution of electron density along the [001] conduction pathway confirming this.^31^ Secondly, interpenetration engineering: Chen *et al.*, in a single-crystal-to-single-crystal transformation from 7-fold to 8-fold interpenetrated MOF, SHG intensity increased by ∼125 times due to enhanced π–π interactions and oscillator strength in the more interpenetrated structure.^178^ Third: guest loading: for cage-like Cu-MOF encapsulated perovskite quantum dot (PeQD) systems, a 6.36-fold enhancement in third-order NLO absorption was achieved compared to bare PeQDs due to charge transfer between the permeating confined QDs and corresponding MOF walls.^22^ MOFs provide unique advantages against competing material classes when assessed in the context of conventional NLO materials. DAST is a widely studied organic push–pull chromophore, however, it suffers from concentration quenching, poor photostability and challenging crystal growth.^8^ Inorganic crystals (KDP, LiNbO_3_), are photostable, but have fixed non-tunable responses and do not permit guest-modulated switching because they are impermeable. MOFs represent a convenient compromise: the rigid lattice minimizes exciton aggregation quenching, reticular chemistry allows linker-mediated tuning of *β*, and their porous architecture allows for post-synthetic modification. The only remaining challenge is producing central-crystal optical quality and phase-matched geometries for device integration.

#### 5.4.10 DFT calculation of hyperpolarizability in MOFs

DFT provides the first hyperpolarizability (*β*) and second hyperpolarizability (*γ*), which are the most vital NLO descriptors at the molecular level. In the case of isolated MOF linker models, coupled-perturbed Kohn–Sham (CPKS) is the most established methodology currently available to calculate static *β*_0_ values using hybrid functionals and polarized basis sets.^179^ It can be recommended that CAM-B3LYP over B3LYP for push–pull D–π–A systems, because the long-range-separated exchange correction of CAM-B3LYP prevents from the widely documented overestimation of *β* in charge-transfer chromophores.^180^ For periodic MOF systems, dielectric tensor and linear optical response are obtained through the CPKS framework in CRYSTAL Schwarz *et al.* (2016), but fully periodic frequency-dependent *β* calculations are still computationally complicated and typically modeled at a cheaper level using linker-fragment models. The experimental validation for MIL-125–NH_2_ has been shown recently, using DFT to confirm that amino-functionalization facilitates a decrease in optical band gap through N 2p electron-donation.^180^ For comparison, here in MoF-bpy a DFT-calculated band gap of 2.53 eV is obtained against an experiment-determined barrier of 2.39 eV (error < −5%),^48^ while for Cu-bpy MOF the reactive barrier determined by first-principles DFT was again accurately reproduced for a calculated band gap of 2.39 eV *versus* experimental value of 2.28 eV (error < 5%)^65^

## 6. Comparative NLO performance across MOF families

Through a detailed cross-family analysis we find that no MOF architecture offers simultaneous enhancement of second- and third-order NLO responses, whilst some inorganic crystals still rival our best examples in certain metrics. For second-order activity (SHG), bimetallic Zn/Cu MOFs currently hold the title for highest *d*_33_ ≈ 19.86 pm V^−1^ (≈51 times higher than KDP [*d*_36_ = 0.39 pm V^−1^] and several times higher than α-quartz, [*d*_11_ = 0.30 pm V^−1^]) ascribed to strain-mediated symmetry breaking at the bimetallic interface.^163^ Tunable SHG over 800–1500 nm has been achieved with non-centrosymmetric lanthanide MOFs (MOF-Er^3+^/Yb^3+^), which allows a spectral flexibility not available in fixed-gap inorganic crystals.^164^ Third-order NLO for Cu-HHTP conductive MOFs reaches *β* = 7.60 × 10^−6^ m W^−1^ at 0 V and increases to 3.84 × 10^−5^ m W^−1^ at 10 V with electrical tunability being an important strength of the MOF *versus* passive molecular chromophores.^31^ Porphyrin-based interpenetrated MOF ZnTPyP-1 reaches a *χ*^(3)^ = 7.73 × 10^−7^ esu with an optical limiting threshold of 3.6 J cm^2^, nearly exceeding almost all reported NLO active single-component inorganic materials at this wavelength (532 nm).^63^ TPA containing MOFs always give *β* values in the 10^−3^ to 10^−4^ cm W^−1^, competing favourably with the benchmark push–pull organic chromophores but offering additional benefits of solid-state processability and forced alignment due to being framework confined. All families share a general trade-off between NLO magnitude and centrosymmetric, with frameworks leading to the largest third-order responses (Cu-HHTP, ZIF-8 composites) largely remaining centrosymmetric in character and thus SHG-silent; large non-centrosymmetric designs tuned for SHG almost universally sacrifice third-order performance. This intrinsic tension grounds the main design strategy challenge in MOF-NLO engineering, and permits the discussion of the hybrid bimetallic and guest@MOF strategies in following section.

## 7. Future challenges and directions

### 7.1 DFT accuracy challenges for MOF-NLO

Although this has led to enormous improvements in DFT based MOF-NLO characterization, there are a number of inherent accuracy issues. The most severe is the systematic under-estimation of band gaps with standard GGA functionals like PBE, which can yield errors of 12 eV or worse than experimental values and especially with MOFs with open-shell 3d transition metal cations.^39^ This underestimation directly carries over to the overestimated NLO responses when GGA-derived band gaps are employed when estimating 2-state models of *β*. Although hybrid functionals (HSE06) can significantly enhance accuracy, they are expensive and large-scale systems (MOFs with small unit cells) cannot be readily studied with them because of their high cost. The second problem is the calculation of dispersive (van der Waals) interactions in MOF geometries, which are important to get the igneous stacking of aromatic linkers right. DFT-D3 dispersion terms are becoming the norm but increase computational cost. Third, the dynamics simulation, such as linker flexibility, thermal motion, and guest-induced distortion, necessitate molecular dynamics simulations beyond static DFT are being actively pursued together with machine learning force field to become less expensive.

### 7.2 High-throughput DFT screening assisted with machine learning

Experimental and even DFT screening MOFs, with millions of possible combinations of linkers and nodes, is intractable without computational acceleration. Machine learning (ML) models trained on DFT-computed property data (including the QMOF Database^10^) are capable of predicting band gaps, optical absorption spectra and NLO-related electronic descriptors of hypothetical MOFs in milliseconds, allowing high-throughput virtual screening of NLO candidates prior to synthesis. Graph neural networks (GNNs) that are trained on MOF topology and chemical properties have a specific likelihood of predicting properties. ML-guided screening combined with focused high-accuracy DFT validation and in the spirit of our rational design philosophy, applied to the design of molecular NLO systems and is the new paradigm of MOF-NLO design.

Due to the combinatorial size of MOF chemical space, it is intractable to even attempt exhaustive DFT screening. Thus, ML models trained on DFT-computed property databases have become an indispensable acceleration layer in MOF-NLO design, with prediction times of milliseconds per structure *versus* thousands of computing hours for a full DFT calculation.^39^ The largest ML-accessible database of MOF electronic properties is the QMOF (for “Quantum MOF”) database of over 14 000 experimentally characterized MOFs with DFT-computed band gaps, frontier orbital energies and optical properties all calculated at a consistent PBE level-of-theory.^181^ As band gap is the most direct electronic descriptor that governs NLO response *via* its inverse dependence on polarizability and hyperpolarizability, this QMOF database serves as an immediate basis for ML-guided screening of NLO candidates.

Various ML architectures have been used for MOF property predictions that are directly relevant to NLO. Mean absolute errors of 0.25–0.35 eV achieved for band gap prediction across the QMOF dataset by graph neural networks (GNNs), which encode MOF crystal structures as atomic graphs and learn structure–property relationships through message-passing layers, are within sufficient accuracy ranges for first-pass NLO screening.^39^ Linker-level NLO descriptor prediction by Achour *et al.*, an extensive dataset of 195 derivatives of diphenyl thioether linkers for NDI were studied with hybrid DFT–LightGBM scheme to build *e.g.* improved HOMO–LUMO gaps prediction ability so that test *R*^2^ = 0.86 and RMSE = 0.25 eV was achieved plus100 µs savings as compared to full DFT.^182^ In that work, topological, electronic and steric descriptors emerged as the most important molecular features controlling gap modulation based on SHAP analysis, yielding chemically interpretable design rules for linker optimization directly relevant to NLO-active MOF design.

As such, the practically implemented ML-guided MOF-NLO design workflow follows the steps: (1) high-throughput screening across a QMOF database based on band gap and charge-transfer energy filters; (2) hyperpolarizability relevant descriptors prediction *via* GNN or gradient boosting for these shortlisted candidates; (3) calculation of full DFT hyperpolarizability on top-ranked structures for further analysis and ranking; (4) experimental validation through synthetic procedures eventually followed by Z-scan or SHG measurements. His hierarchy allows for the design cycle to be compressed from years to weeks and represents a new pathway for next-generation MOF-NLO discovery.

### 7.3 An underexplored frontier: frequency-dependent NLO of MOFs

Surprisingly, whereas there is a wealth of data of MOF band gaps and optical absorption using the exact DFT, frequency-dependent NLO calculation SHG [*β*(−2*ω*, *ω*, *ω*)] and EOPE [*β*(−*ω*, *ω*, 0)] at experimentally relevant laser wavelengths is extremely scarce in the literature of MOF. This is an essential discontinuity, since frequency-dependent *β* values at standard Nd:YAG laser wavelengths (532 nm and 1064 nm) are the main experimental NLO parameters that are measured by Z-scan and Maker fringe techniques. We have repeatedly prepared frequency-dependent NLO properties of all of our molecular systems (532 nm, 1064 nm, 1907 nm) in our own experiment,^183^ which offers a straightforward way to transfer our methodology to the characterization of MOF linkers with NLO. Future directions in work should focus on frequency-dependent DFT computations of MOF linker models, which will allow a direct comparison with experimental MOF and NLO data.

### 7.4 Chiral MOFs to SHG: a design space to explore

Chiral metal–organic frameworks which are synthesized by chiral ligands, or by spontaneous symmetry breaking of achiral building blocks are one of the future directions of SHG active materials since chirality implies NCS crystal structures in the first place. DFT calculations on chiral MOF linkers can compute the molecular hyperpolarizability of the linker and, on top of the crystal field corrections, predict the bulk SHG tensor components. A logical approach to MOF, SHG materials is the development of chiral MOFs using chirality and long π-conjugation NLO-active chromophores, including BINOL-carboxylates, camphoric acid, or amino acid-derived linkers. Lanthanide chiral MOFs are of special interest since the lanthanide ions are optimal to optimize optical activity due to their high spin–orbit interaction and f–f transitions in the NIR.

### 7.5 Guest@MOF NLO switching and ON/OFF response

The porous nature of MOFs makes possible a novel NLO switching paradigm: chromophore guest molecules with large hyperpolarizabilities can be incorporated into MOF pores, and the NLO response can be turned ON or OFF by loading/unloading guests, by photoisomerization, or by redox. DFT analysis of guest@MOF composites and computing summed-up hyperpolarizability of host MOF with NLO guest is a conceptually rich extension of our previous ON/OFF NLO analysis framework.^40^

### 7.6 Nanosheets and thin films of 2D MOF

Another promising technology is the 2D MOF nanosheets that can be deposited on substrates by layering to form oriented thin films, thereby providing very high NLO capabilities with large surface to volume ratios, controlled chromophore orientation, and increased 2D plane π-conjugation. ZnTPyP(Co) 2D MOF thin film exhibited *χ*^(3)^ ≈ 2.63 × 10^−7^ esu,^70^ a value better than most 2D inorganic NLO materials. DFT simulation of 2D MOF electronic structure through periodic slab calculations that paired with the polarizability analysis methods of molecular DFT – is a potent methodology to design next-generation 2D MOF NLO materials. The design parameters that are critical are the in-plane π-conjugation network, NLO-active center density and the stacking order.

## 8. Conclusion

MOFs have emerged over the past decade as a uniquely versatile NLO platform, the present review has critically analyzed the nonlinear optical characteristics of metal–organic frameworks in the complementary perspectives of structural design, experimental characterization and density functional theory computation. The analysis reveals a number of major themes. To begin with, non-centrosymmetric crystal structures are the determining condition to achieve second-order NLO activity (SHG), which can be guided by a combination of wise selection of chiral linkers, asymmetric pairs of donor–acceptor ligands, non-centrosymmetric metal coordination geometries, and bimetallic strain engineering, which has been shown to produce *d*_22_ values (−19.86 pm V^−1^) second, triphenylamine-based MOFs are a sub-class of third-order NLO materials that is especially potent, since the multibranched TPA donor core provides an advantageous response to external modulation strategies like electric-field tuning, guest loading, and interpenetration engineering, which can increase the responses by one to two orders of magnitude. Third, DFT is now necessary to rationalize and predict NLO behavior in MOFs: hybrid functionals (HSE06) give band gaps and optical absorption onsets correctly, dispersion-corrected methods are needed to get the geometry of linkers correct, and now million-fold acceleration in virtual linker screening with *R*^2^ > 0.866 accurate results is achievable by integrating DFT with machine learning. However, there are still major gaps: MOF systems have hardly been calculated at frequencies where such experiments take place using frequency-dependent hyperpolarizability, systematic DFT characterization of MOF-derived nanocarbons to electrocatalytic applications is insufficiently developed, and much more attention should be paid to thin-film processing, optical quality, and long-term photostability to translate laboratory-scale NLO demonstrations to practice. From the diverse MOF families reviewed, it can be seen that NLO properties mapped directly to four general types of practical photonic applications: solid-state optical power limiters (Cu-bpy, ZnTPyP-1), electrically tunable all-optical switches (Cu-HHTP films), NIR frequency-conversion elements and non-contact thermometers (lanthanide MOFs), and multiphoton bio-imaging agents able to co-load drugs based on porphyrin and TPA-based MOFs. Overcoming these barriers by converging reticular chemistry, high-performance spectroscopy, and high-throughput computation will make MOF-based materials a formidable competitor to conventional NLO crystals and molecular chromophores in the next round of photonic technologies, such as optical limiting, all-optical switching, frequency conversion, and integrated optoelectronic systems.

## Abbreviations

## Conflicts of interest

There are no conflicts to declare.

## Data Availability

No primary research results, software or code have been included, and no new data were generated or analyzed as part of this review.
